# Alzheimer’s disease tau is a prominent pathology in LRRK2 Parkinson’s disease

**DOI:** 10.1186/s40478-019-0836-x

**Published:** 2019-11-16

**Authors:** Michael X. Henderson, Medha Sengupta, John Q. Trojanowski, Virginia M. Y. Lee

**Affiliations:** Department of Pathology and Laboratory Medicine, Institute on Aging and Center for Neurodegenerative Disease Research, University of Pennsylvania School of Medicine, 3600 Spruce St, 3rd Floor Maloney, Philadelphia, PA 19104 USA

**Keywords:** Leucine-rich repeat kinase 2, Aggregation, G2019S, Progressive supranuclear palsy, α-Synuclein, Amyloid β

## Abstract

Mutations in leucine-rich repeat kinase 2 (*LRRK2*) are the most common cause of familial Parkinson’s disease (PD). While the clinical presentation of *LRRK2* mutation carriers is similar to that of idiopathic PD (iPD) patients, the neuropathology of *LRRK2* PD is less clearly defined. Lewy bodies (LBs) composed of α-synuclein are a major feature of iPD, but are not present in all *LRRK2* PD cases. There is some evidence that tau may act as a neuropathological substrate in LB-negative *LRRK2* PD, but this has not been examined systematically. In the current study, we examined α-synuclein, tau, and amyloid β (Aβ) pathologies in 12 *LRRK2* mutation carriers. We find that α-synuclein pathology is present in 63.6% of *LRRK2* mutation carriers, but tau pathology can be found in 100% of carriers and is abundant in 91% of carriers. We further use an antibody which selectively binds Alzheimer’s disease (AD)-type tau and use quantitative analysis of tau pathology to demonstrate that AD tau is the prominent type of tau present in *LRRK2* mutation carriers. Abundant Aβ pathology can also be found in *LRRK2* mutation carriers and is consistent with comorbid AD pathology. Finally, we assessed the association of neuropathology with clinical features in *LRRK2* mutation carriers and idiopathic individuals and find that *LRRK2* PD shares clinical and pathological features of idiopathic PD. The prevalence of AD-type tau pathology in *LRRK2* PD is an important consideration for understanding PD pathogenesis and refining clinical trial inclusion and progression criterion.

## Introduction

Parkinson’s disease (PD), PD dementia (PDD), and dementia with Lewy bodies (DLB) represent a spectrum of neurodegenerative diseases [1–5] collectively known as α-synucleinopathies due to the aggregation of α-synuclein into intracellular Lewy bodies (LBs). The presence of LBs in the brainstem is associated with dopaminergic neuron loss and motor dysfunction in those diagnosed with PD [6]. However, up to 80% of patients develop dementia (PDD) during their disease course [7], and this progression is associated with the presence of LBs in cortical areas [8]. Progression to dementia is also associated with increasing tau co-pathology in a pattern that is similar to that seen in Alzheimer’s disease (AD) [4, 9], but with a greater temporal neocortical distribution [10]. Together, these studies have suggested that α-synuclein and tau pathologies are associated with each other in PD-PDD and may directly influence each other or be driven by a common factor to augment the progression of neurodegeneration. Identifying factors that could influence the progression of disease could provide a valuable target for modifying disease trajectory.

While most PD is idiopathic, insights into disease pathogenesis have been gleaned from genetic mutations which elevate the risk of PD. One of the most common genes mutated in PD encodes leucine-rich repeat kinase (*LRRK2*) [11]. The most prevalent mutation in *LRRK2*, p.G2019S, confers a 25 to 42.5% risk of PD [12], with a similar age of onset and disease duration as idiopathic PD [11], suggesting that it may phenocopy idiopathic PD (iPD). Indeed, the most common feature of *LRRK2* PD, as in iPD, is the loss of substantia nigra neurons [13]. While LBs are another feature of some *LRRK2* PD, approximately 21–54% of reported *LRRK2* mutation carriers have no apparent LBs [13, 14], suggesting that some other disease factor may be responsible for the observed clinical disease. A top candidate for this disease factor is tau, since 79% of *LRRK2* mutation carriers have been reported to have some degree of tau pathology [13].

Further support for tau pathology as the neuropathological substrate of the parkinsonism observed in *LRRK2* mutation carriers has come from the identification of progressive supranuclear palsy (PSP)-like tau inclusions observed in several cases [15–18]. However, the PSP-like tau pathology in these cases was mild, and AD-like tau was also a prominent neuropathological feature [17, 18]. Multiple population studies have found that *LRRK2* mutations are very rare in pathologically-confirmed primary tauopathies PSP or corticobasal degeneration (CBD) [19, 20], suggesting that *LRRK2* mutations are primarily associated with PD. What genetic studies do not clarify is whether or not *LRRK2* mutations could drive tau pathology in the context of *LRRK2* PD. It is also not clear if the tau observed in *LRRK2* mutation carriers is PSP tau or AD tau, and if this pathology is sufficient to be classified as the neuropathological substrate of dopaminergic neuron loss.

The current study uses quantitative pathology analysis to determine the type and extent of protein pathologies present in 12 cases with *LRRK2* mutations. In addition to pathological α-synuclein, tau and Aβ staining used for typical neuropathological assessments, we also used an AD tau-selective antibody, GT-38 [21, 22], to investigate the type of tau present in *LRRK2* PD. We find that tau pathology is a prominent feature of *LRRK2* PD, and that this tau pathology is largely AD-type tau. AD tau staging in LRRK2 PD follows a similar distribution to iPD and iPDD and is accompanied by abundant concurrent Aβ pathology in most cases. Further, tau is not an independent disease factor in *LRRK2* PD, but is associated with the degree of α-synuclein pathology and progression to dementia. Together, these results suggest that *LRRK2* PD is similar to iPD in its accumulation of AD type tau. It will be important for future studies to address whether LRRK2 directly influences the development of tau pathology and whether LRRK2 inhibitors affect tau pathology.

## Materials and methods

### Selection of cases

*LRRK2* mutation carriers with available brain tissue were identified among deceased individuals with *LRRK2* genotyping results at the Center for Neurodegenerative Disease Research (CNDR) at the University of Pennsylvania. Ten individuals were identified who carried the p.G2019S mutation, two of whom were homozygous for this mutation. Three of these carriers were previously described [23]. Two other individuals had been previously described [24] who had variants of unknown pathogenicity. While genotyping has historically been applied on a project-by-project basis, we assessed the likelihood of ascertainment bias in our database by evaluating the percentage of all cases for which *LRRK2* had been genotyped for at least the most common p.G2019S mutation. Remarkably, only 76/1484 (5.1%) of all cases for which DNA was available had not been genotyped, making it unlikely that patients with *LRRK2* mutations were missed due to a lack of *LRRK2* genotyping.

### Immunohistochemistry

Brains were removed and regions of interest were dissected and fixed in either neutral buffered formalin (NBF) or 70% ethanol, 150 mM sodium chloride. Tissue was infiltrated with paraffin, embedded in blocks, cut into 6 μm sections and mounted on glass slides. Slides were then stained using standard immunohistochemistry as described below. Slides were de-paraffinized with 2 sequential 5-min washes in xylenes, followed by 1-min washes in a series of descending ethanol concentrations: 100, 100, 95, 80, 70%. Slides were then incubated in deionized water for one minute prior to antigen retrieval as noted. After antigen retrieval, slides were incubated in 5% hydrogen peroxide in methanol to quench endogenous peroxidase activity. Slides were washed for 10 min in running tap water, 5 min in 0.1 M Tris, then blocked in 0.1 M Tris/2% fetal bovine serum (FBS). Slides were incubated in primary antibodies overnight. The following primary antibodies were used. For pathologically-phosphorylated α-synuclein, pS129 α-synuclein (EP1536Y; Abcam ab51253, RRID:AB_869973) was used at 1:20,000 with microwave antigen retrieval using citric acid-based antigen unmasking solution (Vector H-3300, RRID:AB_2336226). For phosphorylated tau, AT8 (pS202/T205; Thermo Fisher MN1020, RRID:AB_223647) was used at 1:10,000 without retrieval. To selectively detect AD tau, GT-38 (CNDR, [21]) was used at 1:1000 without antigen retrieval. For Aβ, NAB228 (CNDR, RRID:AB_2314850) was used at 1:10,000 with formic acid antigen retrieval.

Primary antibody was rinsed off with 0.1 M Tris for 5 min, then incubated with goat anti-rabbit (Vector BA1000, RRID:AB_2313606) or horse anti-mouse (Vector BA2000, RRID:AB_2313581) biotinylated IgG in 0.1 M Tris/2% FBS 1:1000 for 1 h. Biotinylated antibody was rinsed off with 0.1 M Tris for 5 min, then incubated with avidin-biotin solution (Vector PK-6100, RRID:AB_2336819) for 1 h. Slides were then rinsed for 5 min with 0.1 M Tris, then developed with ImmPACT DAB peroxidase substrate (Vector SK-4105, RRID:AB_2336520) and counterstained briefly with Harris Hematoxylin (Fisher 67–650-01). Slides were washed in running tap water for 5 min, dehydrated in ascending ethanol for 1 min each: 70, 80, 95, 100, 100%, then washed twice in xylenes for 5 min and coversliped in Cytoseal Mounting Media (Fisher 23–244-256).

Finally, slides were scanned into digital format on a Lamina scanner (Perkin Elmer) at 20x magnification so the digitized slides could be analyzed by quantitative pathology.

### Quantitative pathology

All annotation and quantification was done blinded to disease and genotype. All quantitation was performed in HALO quantitative pathology software (Indica Labs). After annotation of all sections for a given stain, all sections were analyzed simultaneously using the same optical density threshold. Optical density thresholds were empirically determined to not include any background signal and were as follows: EP1536Y (0.192), AT8 (0.355), GT-38 (0.213) and NAB229 (0.244). This signal was then normalized to the total tissue area and values were expressed as percentage area occupied by the immunostained pathologies. A minimal tissue optical density of 0.02 was used to exclude any areas where tissue was damaged.

### Statistical analysis

Linear regressions were all performed in R (https://www.R-project.org/) [25] as described in the figure legends and results section, and the results of these tests are reported in the figure legends. Comparisons of pathology in subgroups (non-demented versus demented; pSyn positive versus pSyn negative) brains was done in GraphPad Prism 7 and results are reported in the figure legends.

## Results

### Prevalence of α-synuclein pathology in *LRRK2* mutation carriers

Previous studies have described heterogeneous pathologies in *LRRK2* mutation carriers, depending on the areas surveyed and antibodies used [13, 14]. In the current study, all autopsied cases in our brain bank with a documented *LRRK2* variant were selected for quantitative pathology analysis. We present 12 cases: 10 p.G2019S variant carriers and 2 rare variants with unclear *LRRK2* pathogenicity—p.L1165P and p.R793M. The two rare variants were previously described as possibly pathogenic [24], although subsequent research also found p.R793M in two healthy individuals [26], suggesting that R793M may be a rare polymorphism. Of the 10 p.G2019S carriers, 7 had a clinical diagnosis of PD, 2 had a clinical diagnosis of PDD, and 1 had a history of schizophrenia. While the schizophrenia case was included in histological analyses, it was not included in subsequent determination of pathology prevalence since the patient did not suffer from neurodegenerative disease. Both of the rare variant cases had a clinical diagnosis of PDD.

**Fig. 1:**
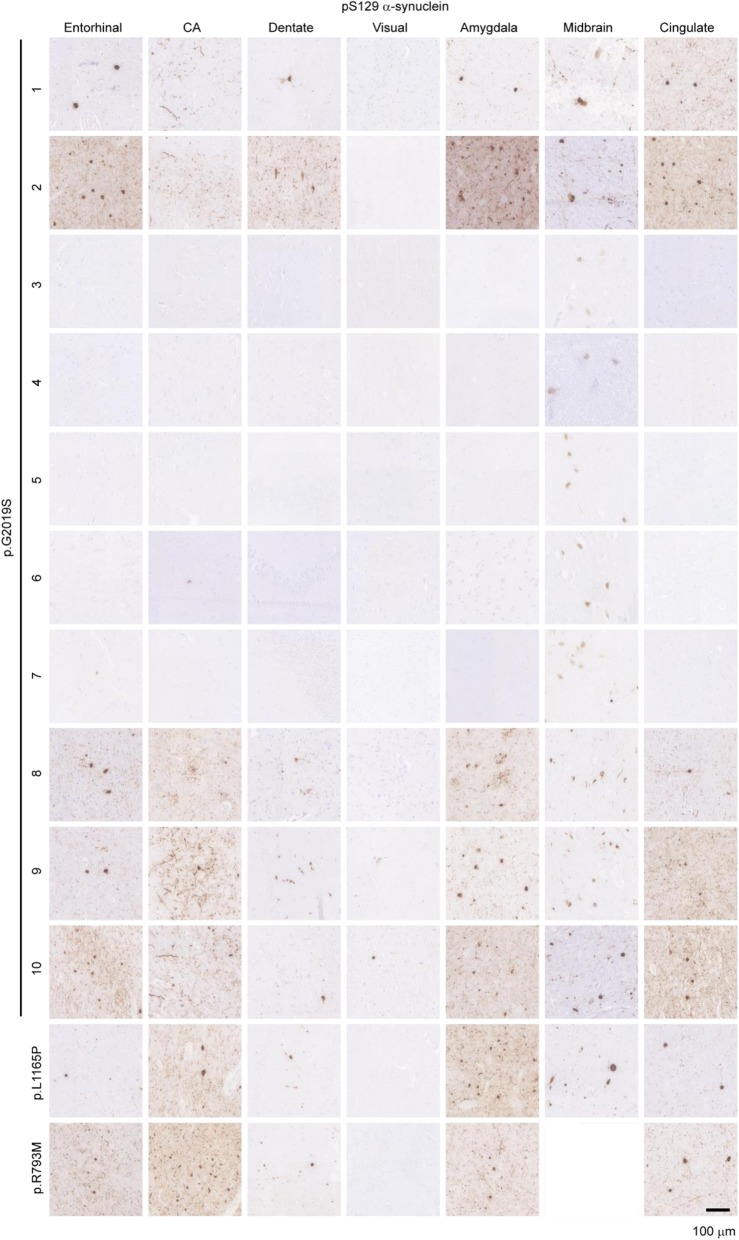
Prevalence of α-synuclein pathology in *LRRK2* mutation carriers. Seven brain regions from 12 individuals carrying *LRRK2* mutations were evaluated by staining for pathological pS129 α-synuclein (EP1536Y). Of the 12 cases, 7 showed prominent pathological α-synuclein throughout the brain, including the substantia nigra, while 5 cases had minimal pathological α-synuclein present. Scale bar = 100 μm

Seven brain regions (entorhinal cortex, Cornu Ammonis (CA) regions, dentate gyrus, visual cortex, amygdala, midbrain, and cingulate cortex) were selected to encompass regions that typically accumulate α-synuclein LBs, tau neurofibrillary tangles (NFTs), other tau inclusions, and Aβ plaques. We first examined the prevalence of α-synuclein pathology using an antibody targeting pS129 α-synuclein (pSyn, EP1536Y, Fig. 1). Excluding the schizophrenia case (p.G2019S-5), which had no pathology of any type, 63.6% (7/11) of cases showed prominent pSyn pathology, including 55.5% (5/9) of the p.G2019S cases. pSyn pathology was abundant in the substantia nigra, amygdala, hippocampus and cingulate cortex in cases where it was found and completely absent in the 5 negative cases. While α-synuclein pathology is typically a necessary neuropathological criterion for PD diagnosis, a small percentage of cases present clinically with PD and have nigral degeneration, but do not have LBs or another pathology to which the neuron loss can be ascribed. We found that 8.3% (9/109) of all iPD cases or 3.7% (9/242) of the iPD-PDD cases in our database met these criteria and therefore were classified as “atypical PD,” similar to the *LRRK2* mutation carriers lacking LBs.

### Prevalence of tau pathology in *LRRK2* mutation carriers

**Fig. 2:**
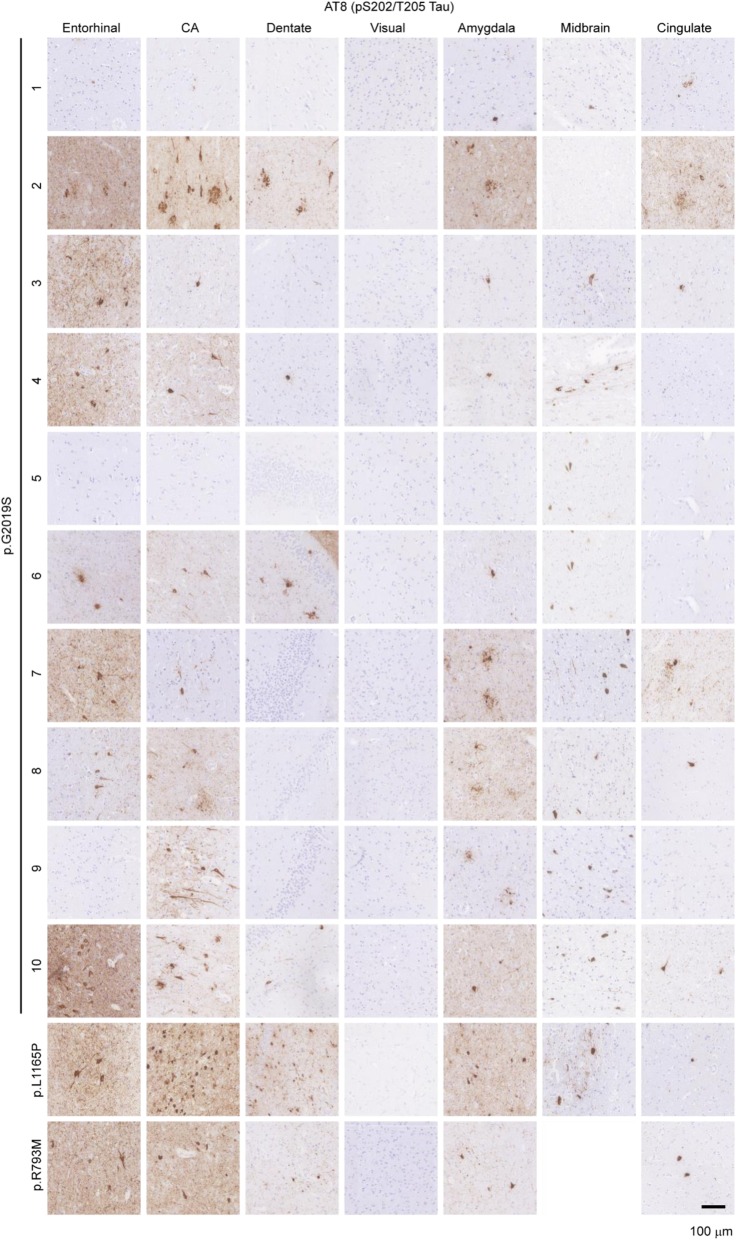
Prevalence of tau pathology in *LRRK2* mutation carriers. Seven brain regions from 12 individuals carrying *LRRK2* mutations were evaluated by staining for pathological pS202/T205 tau (AT8). Of the 12 cases, 10 showed prominent pathological tau, including in the hippocampus, while 2 cases had minimal pathological tau present. Scale bar = 100 μm

Next, we sought to determine the prevalence of tau pathology in *LRRK2* mutation carriers using a pathogenic (pS202/T205) anti-tau antibody (AT8, Fig. 2). Surprisingly, 100% (11/11) cases had some tau pathology, including neurofibrillary tangles, neuritic plaques and neuropil threads, with only one PD case (p.G2019S-1) having rare inclusions. The schizophrenia case (p.G2019S-5) had no pathology. Areas with prominent pathology included the amygdala, hippocampus, and entorhinal cortex. The abundant hippocampal pathology in most cases and inclusion morphology suggest that the tau in these cases is AD-type tau, but we sought to unambiguously characterize the type of tau using an AD-selective anti-tau antibody.

### GT-38 selectively detects AD tau, but not PSP, Pick’s or CBD pathological tau

Our lab has recently generated a series of monoclonal antibodies against an AD tau immunogen [21]. Many of these antibodies showed conformational selectivity for misfolded tau over monomeric tau, and intriguingly, two antibodies showed selectivity for AD tau over PSP, CBD or Pick’s disease tau. This selectivity seems to arise from the presence of both 3 and 4 microtubule-binding repeat isoforms (3R and 4R, respectively) in the tau aggregates as seen in AD. The other tauopathies are primarily composed of a single repeat isoform: PSP (4R), CBD (4R), and Pick’s (3R). This allows the selective detection of AD tau pathology only, even in the presence of abundant non-AD tau inclusions [21, 22].

**Fig. 3:**
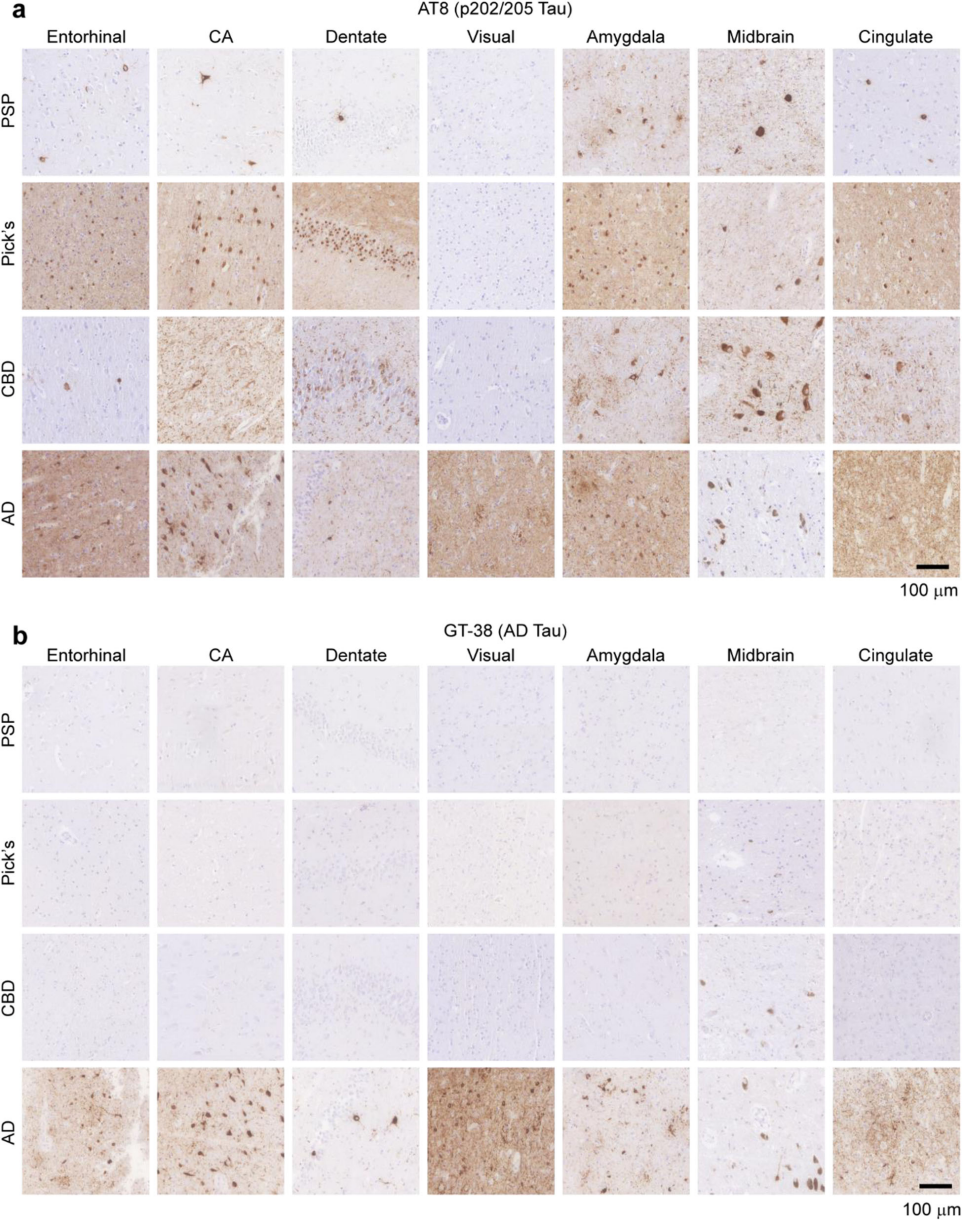
GT-38 selectively detects AD tau, but not PSP, Pick’s or CBD pathological tau**. a** AT8 was used to stain pathological tau in 7 regions from 4 different tauopathy brains, PSP, Pick’s disease, CBD and Alzheimer’s disease AD. Consistent with their neuropathological designation as tauopathies, all four diseases had prominent AT8 staining. **b** GT-38 was used to stain adjacent sections to those stained in panel (**a**). GT-38 staining is absent in the PSP, Pick’s and CBD tissue, despite the high degree of pathogenic tau. In contrast, GT-38 shows prominent staining in all regions of the AD brain. Scale bar = 100 μm

We first confirmed the selectivity of one of these antibodies (GT-38) in PSP, Pick’s, CBD, and AD cases (Fig. 3). Staining of these cases with AT8 shows abundant tau pathology in all four cases and in various regions (Fig. 3a), consistent with presentation of these diseases. GT-38, in contrast, shows no staining of PSP, Pick’s, or CBD tau, but prominent staining of AD tissue (Fig. 3b). It should be noted that while GT-38 only recognizes AD tau, it seems to recognize a subset of that pathology, resulting in reduced overall staining (See Fig. 3b, especially entorhinal cortex and amygdala). Pathological α-synuclein and Aβ staining in these cases can be found in Additional file 1: Figure S1.

### Prevalence of AD tau pathology in *LRRK2* mutation carriers

**Fig. 4:**
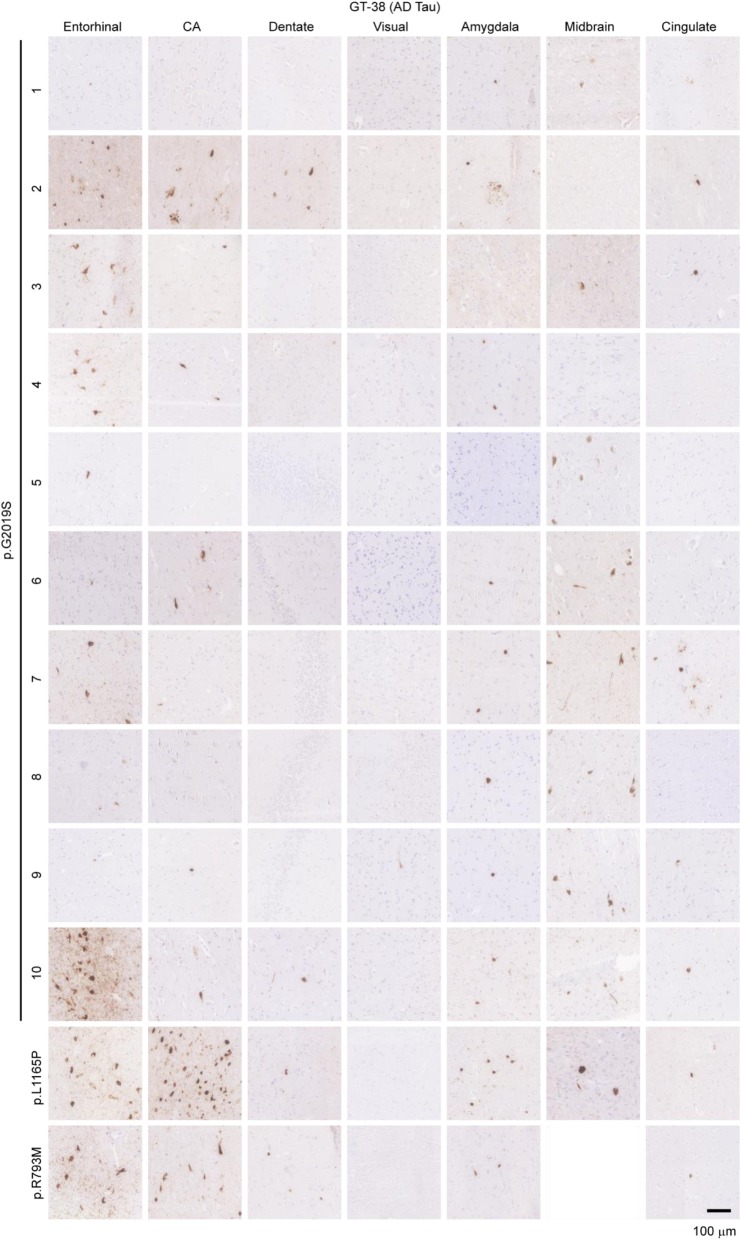
Prevalence of AD tau pathology in *LRRK2* mutation cases**.** Seven brain regions from 12 individuals carrying *LRRK2* mutations were evaluated by staining for pathological AD tau (GT-38). Of the 12 cases, most had apparent AD tau, albeit to different degrees. Scale bar = 100 μm

In order to determine whether the tau pathology observed in *LRRK2* mutation carriers was indeed AD tau, adjacent sections to those stained with AT8 were stained with GT-38 (Fig. 4). As previously noted, GT-38 staining is not as strong, even in pure AD tissue, but all regions that were recognized by AT8 were also recognized by GT-38. Therefore, tau inclusions observed in *LRRK2* mutation carriers are consistent with AD tau pathology. Aβ pathology was also abundant in most of the *LRRK2* mutation carriers, consistent with comorbid AD pathogenesis (Additional file 1: Figure S2). Intriguingly, the two rare variant cases, p.L1165P and p.R793M had no Aβ pathology, despite having abundant AD tau. This finding is explored further in the discussion.

### Quantitative pathology to understand pathological contributions to disease presentation

**Fig. 5:**
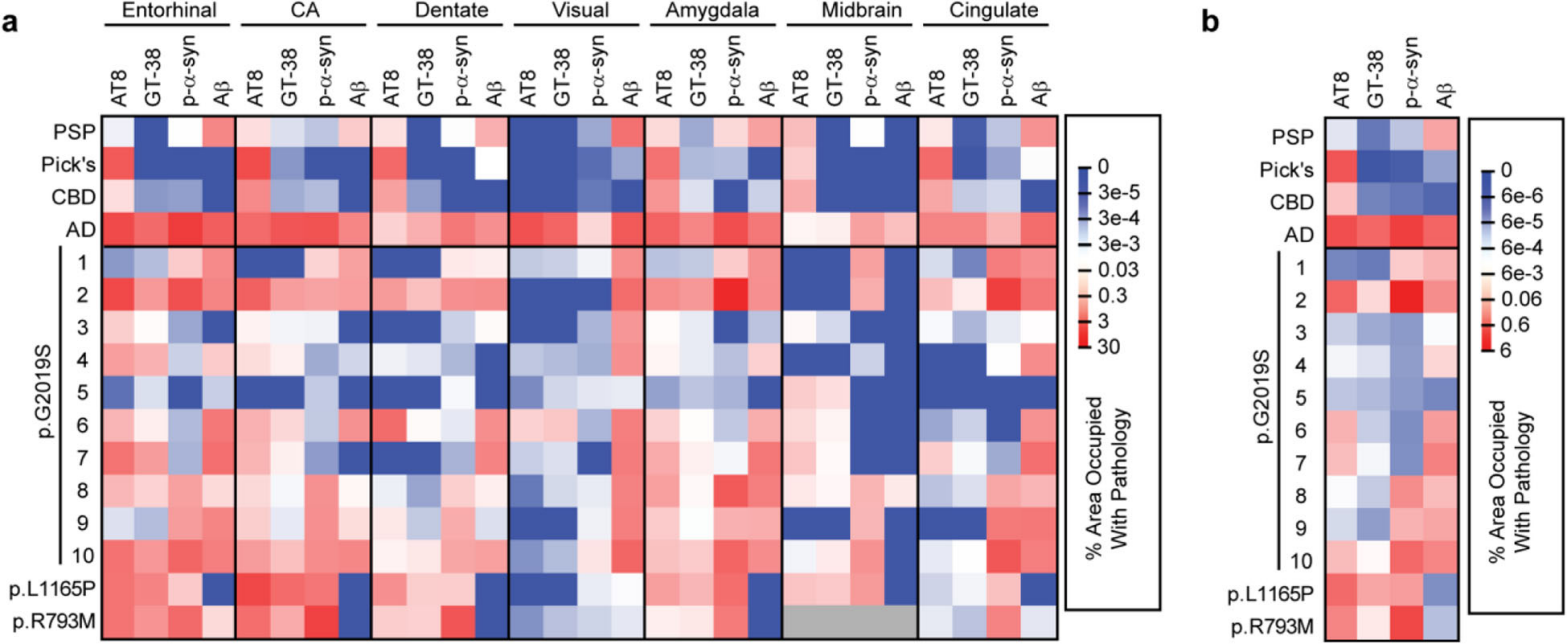
Quantitative pathology heat maps from *LRRK2* mutation carriers and control tauopathy cases**. a** The percentage area occupied with pathology using four different antibodies which reveal total tau pathology (AT8), AD tau pathology (GT-38), α-synuclein pathology (p-α-syn) or Aβ pathology (Aβ) is shown here as a heat map. Warm colors represent regions with high pathology, while cool colors represent regions with low or no pathology. The gray color represents a region that was not available for staining. The values used to generate this table are available in Additional file 2: Table S1. **b** The average percentage area occupied for all regions examined is displayed here as a heat map, allowing rapid examination of overall pathology patterns in all cases

While pathology observations allowed us to make general conclusions about the pathology in *LRRK2* PD cases, we sought a greater understanding of pathology and associations with clinical measures through quantitative pathology. All sections were digitized and gray matter regions were manually annotated in HALO software for automated quantitation. Once annotated, algorithms were developed that thresholded pathology detection by optical density. Thresholds were optimized for each immunohistochemical stain to provide broad detection of pathology in regions with both high and low density pathology, without picking up any background stain (Additional file 1: Figure S3). Pathology values are reported as the percentage of area occupied, and ranged over several log-fold (Fig. 5). Pathology values both by region (Fig. 5a) and mean pathology by case (Fig. 5b) are reported (Additional file 2: Table S1) and were used for subsequent analyses.

### *LRRK2* mutation carriers have AD-type tau pathology

**Fig. 6:**
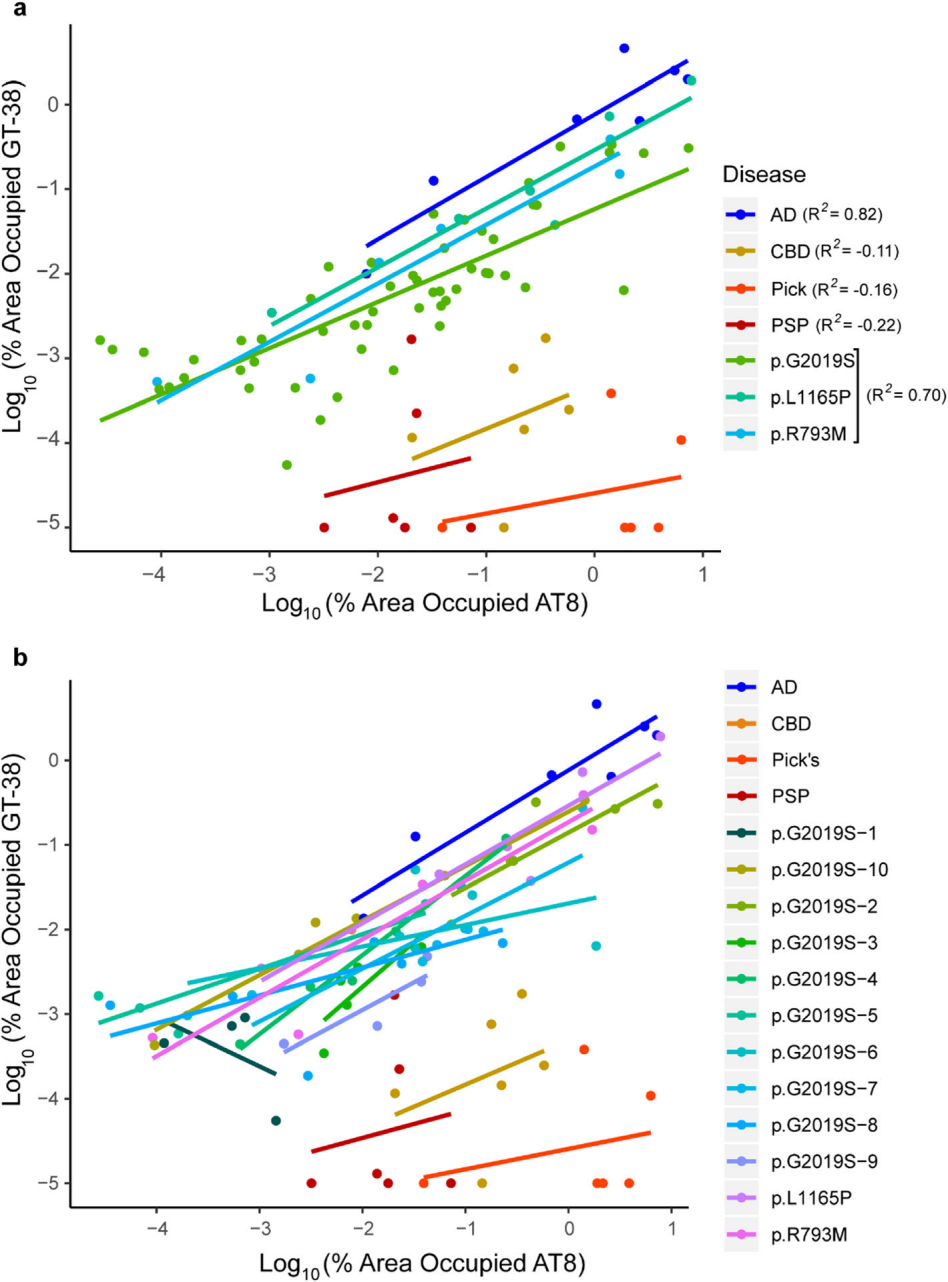
*LRRK2* mutation carriers have AD-type tau pathology. **a** The log value of AT8 and GT-38 pathology for each region is plotted as a scatterplot, with different diseases or genotypes represented by different colors. Linear regression best-fit lines are shown in the same colors. AD and *LRRK2* mutation carriers all show high correlation between AT8 and GT-38 values (AD: *R*^*2*^_*adj*_ = 0.82, *p* = 0.002946; *LRRK2* mutation: *R*^*2*^_*adj*_ = 0.70, *p* < 2.20e-16), suggesting that the predominant tau form is AD tau. In contrast, AT8 and GT-38 values show no correlation in CBD, Pick’s and PSP cases, resulting in segregation of these regions from *LRRK2* mutation carriers and AD cases (CBD: *R*^*2*^_*adj*_ = − 0.11, *p* = 0.5137; Pick’s: *R*^*2*^_*adj*_ = − 0.16, *p* = 0.6037; PSP: *R*^*2*^_*adj*_ = − 0.22, *p* = 0.7743). **b** The same regional AT8 and GT-38 pathology values as in panel (**a**) are plotted here, but with each different case plotted as a different color and with linear regression done on each case independently. All but one of the *LRRK2* mutation carriers overlays nicely, suggesting that each case independently has AD tau. The one exception, p.G2019S-1 has very minimal tau pathology and therefore cannot be described as having AD tau

To understand quantitatively the degree to which the tau pathology observed in *LRRK2* mutation carriers was AD tau, the percentage area occupied with GT-38 pathology was plotted against the AT8 pathology burden in the same areas (Fig. 6). Cases with primarily AD tau are expected to show a good correlation between the two pathology measures, while primary tauopathies would not. Indeed, the AT8 and GT-38 measures correlated very well in the AD case, but not in CBD, Pick’s or PSP cases (Fig. 6a). The p.G2019S, p.L1165P and p.R793M cases all clustered with the AD case, but not with the other tauopathy cases, suggesting that the majority of tau in these cases is AD tau. To further investigate the type of tau on a case-by-case basis, linear regressions were fit individually for each case (Fig. 6b). *LRRK2* mutation carrier cases all continued to cluster and show positive correlation between AT8 and GT-38, with the exception of p.G2019S-1. This case has minimal tau pathology (Figs. 2, 4), and all points clustered in the bottom left of the graph, with an overall negative correlation. Even this case was distinct from the high AT8, low GT-38 clusters of CBD, Pick’s, and PSP cases.

While on average *LRRK2* mutation carriers clustered with AD pathology, it is possible that some regions had non-AD pathology that was obscured by the high AD pathology in other regions. Having quantitative AT8 and GT-38 pathology measures provides the unique opportunity to examine regions in an unbiased manner for potential non-AD pathology. Regions that fell closer to the CBD, Pick’s and PSP clusters were more closely examined to determine whether any substantial non-AD tau was present. Several regions showed substantial AT8 immunoreactivity, but minimal GT-38 immunoreactivity (Additional file 1: Figure S4). Interestingly, these regions did not appear to have non-AD tau, but instead abundant neuritic plaques and low neurofibrillary tangles. This pattern is consistent with the preferential recognition by GT-38 of tangle pathology, and is a caveat of using this stain to rule out the presence of non-AD tau. Using this strategy, we were unable to identify substantial non-AD tau in the regions and cases surveyed.

### Rare tau pathology in the midbrain is mostly AD-type tau

**Fig. 7:**
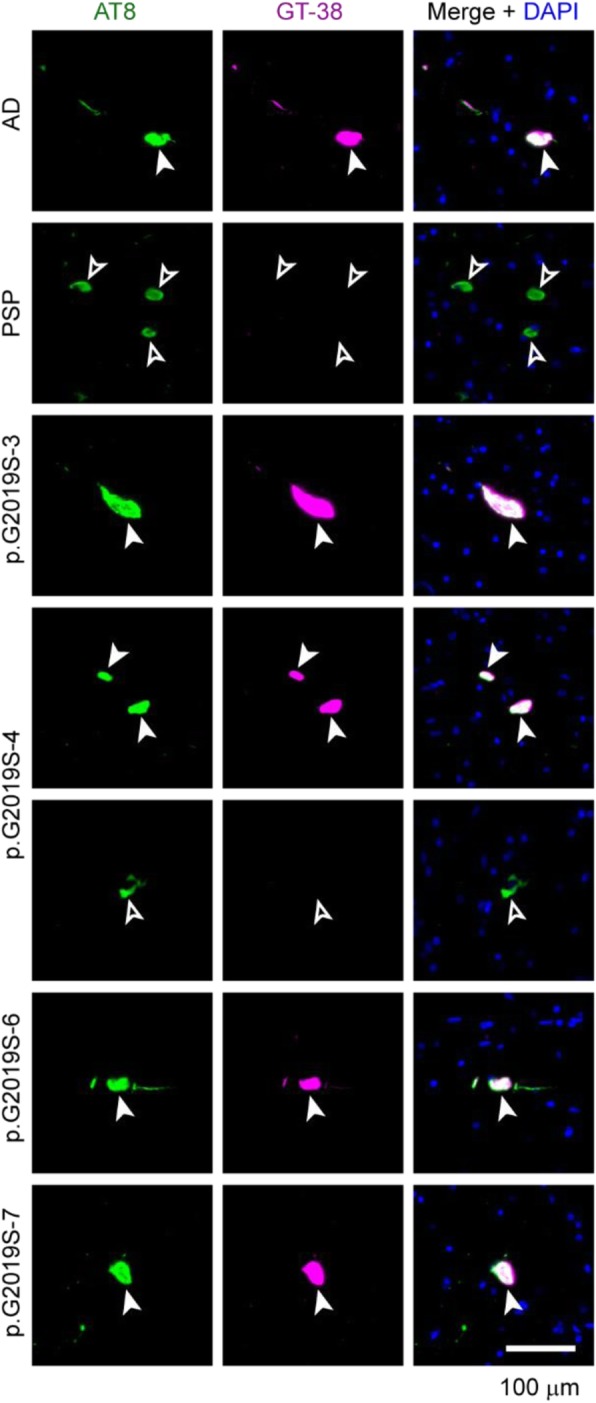
Rare tau pathology in the midbrain is mostly AD-type tau. To further examine whether tau pathology in the midbrain could contribute to neuron loss, midbrain sections from the AD control, PSP control, or LB-negative *LRRK2* mutation carriers were labeled with AT8 (green) for total tau pathology and GT-38 (magenta) for AD tau pathology. The AD case had tau pathology in the nigra, most of which was positive for both AT8 and GT-38 (filled arrowheads). The PSP midbrain contained abundant AT8-positive inclusions that were not recognized by GT-38 (empty arrowheads). Almost all tau inclusions in the *LRRK2* mutation carriers examined were also GT-38 positive, with the exception of one inclusion identified in p.G2019S-4, which was GT-38 negative. Scale bar = 100 μm

One of the questions we sought to address is whether tau is the neuropathological substrate of neuron loss in the substantia nigra of *LRRK2* mutation carriers who lack LB pathology (p.G2019S-3, 4, 6, 7). Each of these cases showed minimal tau inclusions in the midbrain by both AT8 and GT-38 independently, suggesting that tau is not likely to be causing neuron loss in this region (Fig. 2). However, midbrain tau pathology is more often associated with non-AD tauopathies, so we chose to investigate the type of tau present in these cases more closely. Co-immunofluorescence of AT8 and GT-38 in sections from the midbrains of LB-negative *LRRK2* mutation carriers was used to assess the type of tau in these cases (Fig. 7). The AD and PSP cases were used as controls. Tau in the AD midbrain was mostly recognized by both AT8 and GT-38, suggesting that a similar strain of tau is present throughout the brain of this AD case. In contrast tau in the PSP midbrain was AT8-positive, but showed no immunoreactivity with GT-38. The sparseness of tau pathology in the midbrain of *LRRK2* mutation carriers was confirmed in this experiment, but intriguingly most of the tau inclusions in these brains were recognized by both AT8 and GT-38, suggesting that it is predominantly AD tau. One GT-38-negative tau inclusion was observed in p.G2019S-4, but this type of inclusion was rare.

### Tau pathological burden is related to α-synuclein pathology in *LRRK2* mutation carriers

**Fig. 8:**
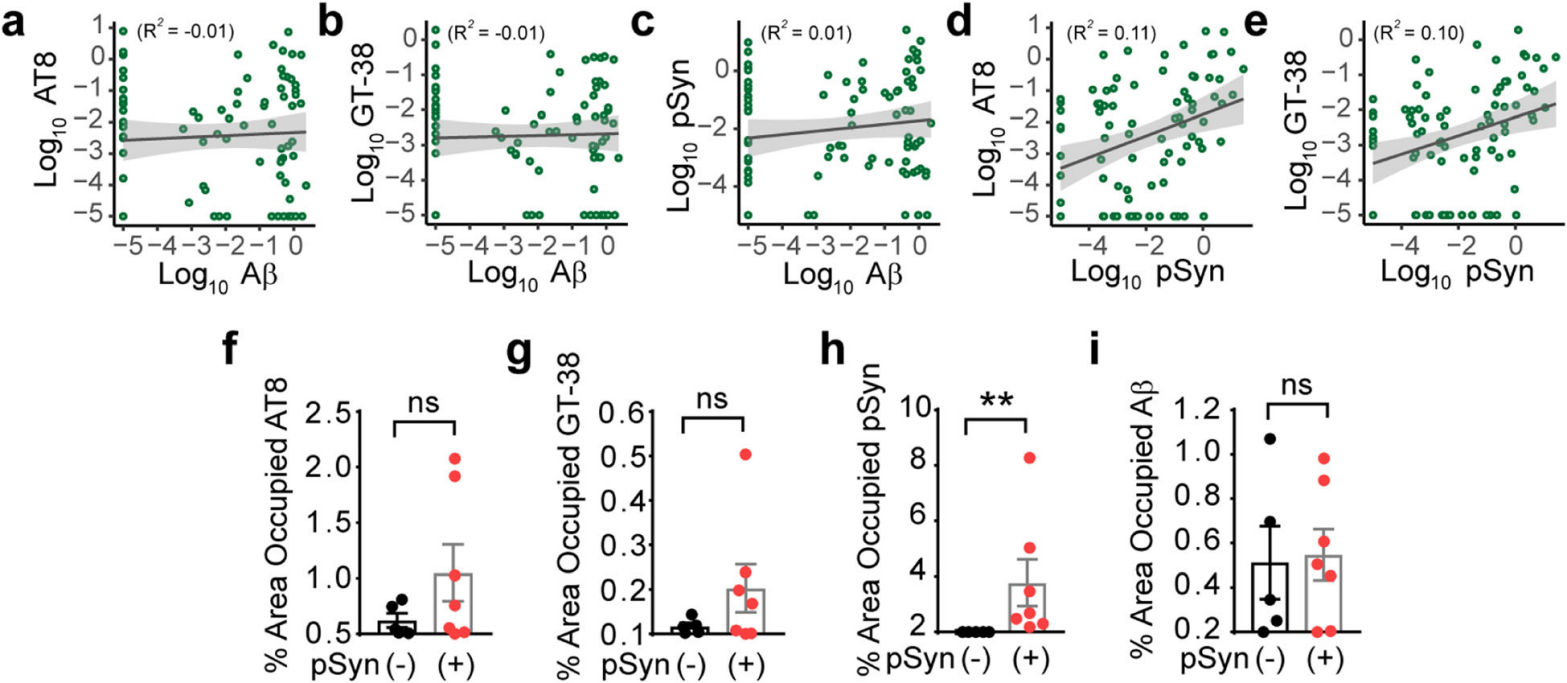
Tau pathological burden is related to α-synuclein pathology in *LRRK2* mutation carriers. Many regions of the brain had multiple pathologies co-occurring. To understand whether there is an association of the different pathologies with each other, each pathology was plotted against the other by region (**a-e**). While Aβ burden showed no relationship to tau or α-synuclein burden (**a** Aβ x AT8: *R*^*2*^_*adj*_ = − 0.01, *p* = 0.6054; **b** Aβ x GT-38: *R*^*2*^_*adj*_ = − 0.01, *p* = 0.7676; **c** Aβ x pSyn: *R*^*2*^_*adj*_ = 0.01, *p* = 0.2091), pSyn pathology was positively associated with tau pathology (**d** pSyn x AT8: *R*^*2*^_*adj*_ = 0.11, *p* = 0.001261; **e** pSyn x GT-38: *R*^*2*^_*adj*_ = 0.10, *p* = 0.002507), suggesting that the two pathologies may influence each other or may both be influenced by a common factor. To further parse out whether tau pathology was a separate neuropathological substrate of disease in cases without α-synuclein pathology, cases were separated by pSyn pathology (**f-i**). Of the 12 *LRRK2* mutation carriers, 5 of them clearly had no pSyn pathology present in any of the areas examined. AT8 (**f**), GT-38 (**g**) and pSyn (**h**) pathology was on average elevated in the brains of individuals with pSyn pathology, although only the pSyn levels were statistically different. In contrast, Aβ load was not different between the two groups (**i**). (**f-h**) Mann-Whitney tests: AT8: *p* = 0.3434; GT-38: *p* = 0.5303; pSyn: *p* = 0.0025. (**i**) Unpaired t-test: Aβ: *p* = 0.8598

While *LRRK2* PD resembles idiopathic PD in many ways, the presence of a substantial percentage of cases without α-synuclein pathology has led to some confusion about the neuropathological substrate of disease in these mutation carriers. Our broad survey of brain regions in *LRRK2* mutation carriers has shown that α-synuclein pathology is absent not only in the substantia nigra, but throughout the brain. This suggests that LBs are not simply cleared from this region after neuron death, but that LBs were likely not ever present in these brains. This discrepancy is one of the major reasons tau has been investigated as a neuropathological substrate of disease in these cases. If tau is indeed responsible for motor symptoms in these patients, higher tau pathology might be expected in LB-negative cases. However, we found that while none of the co-pathologies is associated with Aβ pathology (Fig. 8 a-c; Additional file 1: Figure S5a-c), both AT8 and GT-38 tau pathology burden are positively correlated with α-synuclein pathology (Fig. 8 d, e; Additional file 1: Figure S5d, 5e), suggesting that tau and α-synuclein pathology burden are associated. This association could be due to a common factor precipitating both pathologies, the two pathologies enhancing each other indirectly, or the misfolded proteins directly cross-seeding each other. To assess cross-seeding, cases which had both α-synuclein and tau pathologies were visualized by co-immunofluorescence (Additional file 1: Figure S6). While both pathologies were abundant in many of the same regions, they were rarely in the same cell, suggesting that the association of these two pathologies is not through direct cross-seeding.

To further investigate tau pathology burden specifically in pSyn-positive or pSyn-negative cases, average pathology was grouped by pSyn pathology level (Fig. 8 f-i). Contrary to tau acting as an independent neuropathological substrate, tau pathology was higher on average in cases with pSyn co-pathology (Fig. 8 f-h). However, pSyn pathology was not the only indicator of tau burden, since some cases without pSyn pathology had moderate tau pathology and some cases with pSyn pathology had low tau pathology. Aβ pathology was variable and had no relationship with pSyn pathology (Fig. 8i).

### Pathological correlates of clinical features in *LRRK2* mutation carriers

**Fig. 9:**
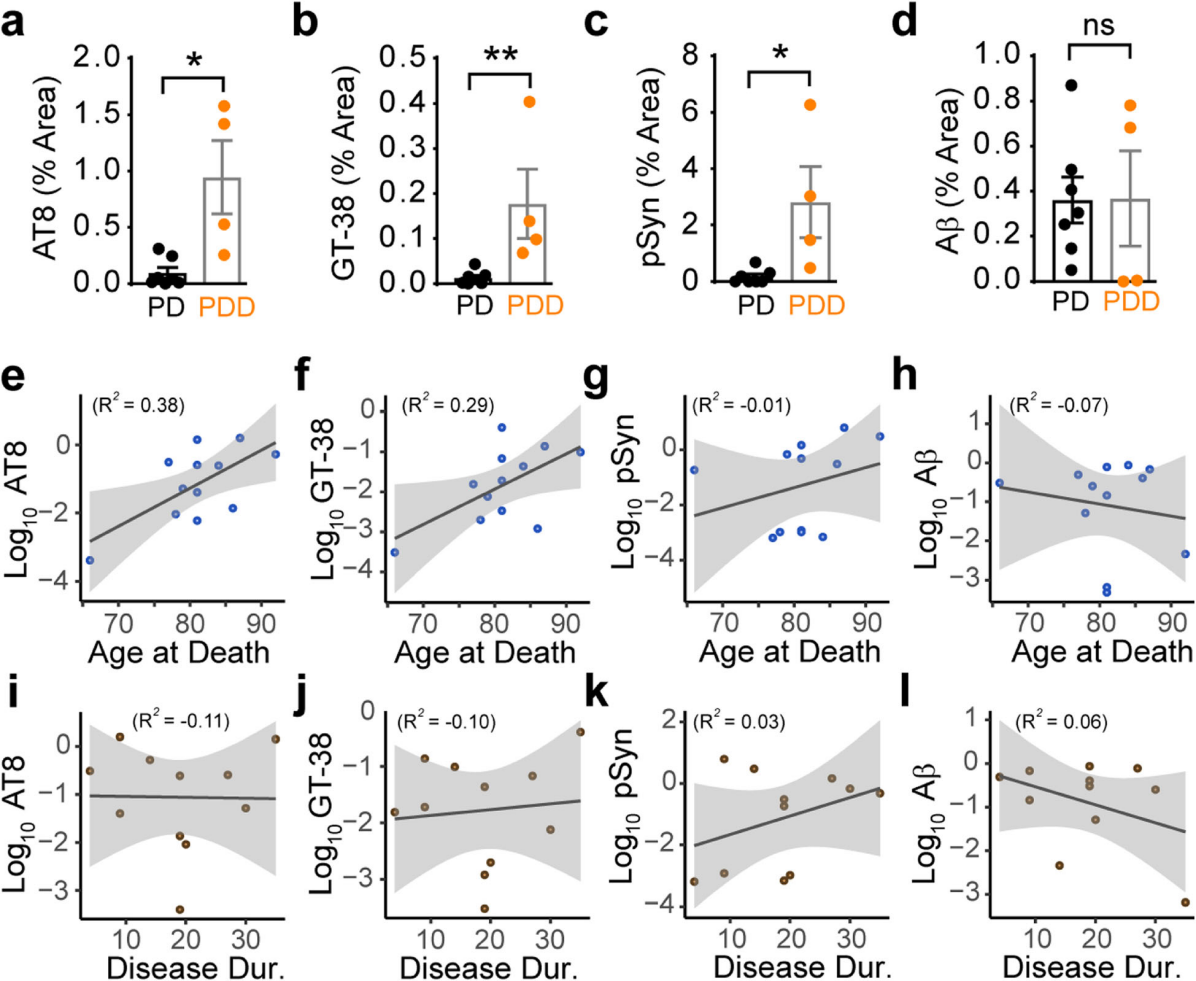
Pathological correlates of clinical features in *LRRK2* mutation carriers. **a-d** Comparisons of pathology levels in *LRRK2* mutation carriers that had either PD or PD with dementia (PDD). Of the 12 *LRRK*2 mutation carriers, 11 of them were characterized clinically as having PD, or having PD followed by dementia. One case had a clinical history of schizophrenia, not PD, and so was removed from this analysis. AT8 (**a**), GT-38 (**b**) and pSyn (**c**) pathologies were significantly elevated in the brains of individuals with dementia. In contrast, Aβ load was not different between the two groups (**d**). **a-c** Mann-Whitney tests: AT8: *p* = 0.0121; GT-38: *p* = 0.0061; pSyn: *p* = 0.0121. **d** Unpaired t-test: Aβ: *p* = 0.9764. **e-h** Mean log_10_ pathology levels were plotted against age at death. AT8 and GT-38 showed the best correlation with age. Lines represent linear regression line of best-fit and shaded area is the 95% confidence interval (AT8: *R*^*2*^_*adj*_ = 0.38, *p* = 0.02012; GT-38: *R*^*2*^_*adj*_ = 0.29, *p* = 0.03969; pSyn: *R*^*2*^_*adj*_ = − 0.01, *p* = 0.3625, Aβ: *R*^*2*^_*adj*_ = − 0.07, *p* = 0.6079). **i-l** Mean log_10_ pathology levels were plotted against disease duration. No pathology measure showed a statistically significant correlation with disease duration (AT8: *R*^*2*^_*adj*_ = − 0.11, *p* = 0.9612; GT-38: *R*^*2*^_*adj*_ = − 0.10, *p* = 0.7663; pSyn: *R*^*2*^_*adj*_ = 0.03, *p* = 0.2791, Aβ: *R*^*2*^_*adj*_ = 0.06, *p* = 0.2386)

*LRRK2* mutation carriers have been reported to have a more prominent tremor [27] and slightly slower disease progression [18], although the presentation of disease is largely overlapping with iPD. In a recent clinicopathological survey of 37 *LRRK2* mutation carriers, progression to dementia was associated with elevated LB pathology [14]. We therefore sought to determine how pathological measures were related to clinical features, including progression to dementia, age at death, and disease duration. Of the 12 *LRRK2* mutation carriers in our cohort, 11 presented clinically with PD, and 4 of those developed dementia over the disease course. Previous research has found that this progression to dementia in idiopathic patients is associated with elevated α-synuclein and tau pathology, especially in cortical regions [4, 9]. *LRRK2* mutation carriers show a similar pattern, with elevated AT8 (Fig. 9a), GT-38 (Fig. 9b) and pSyn (Fig. 9c) in carriers with dementia. In contrast, Aβ levels in these patients showed no association with dementia (Fig. 9d). While age at death (Additional file 1: Figure S7a), age of disease onset (Additional file 1: Figure S7b), and disease duration (Additional file 1: Figure S7c) showed no significant association with dementia, there was a positive correlation between age at death and AT8 (Fig. 9e, Additional file 1: Figure S7e), GT-38 (Fig. 9f, Additional file 1: Figure S7f), and pSyn (Fig. 9g, Additional file 1: Figure S7 g) pathologies. This suggests that tau and α-synuclein pathologies increase with age, but additional factors are responsible for the elevated pathology in individuals with dementia. Aβ pathology showed a negative correlation with age (Fig. 9h, Additional file 1: Figure S7 h). Tau and Aβ pathologies were not associated with disease duration (Fig. 7i, 9j, l, Additional file 1: Figure S7i, 7j, 7 l), and pSyn showed mild positive correlation with disease duration (Fig. 9k, Additional file 1: Figure S7k).

### AD tau staging in *LRRK2* mutation cases compared to idiopathic Lewy body disorders

**Fig. 10:**
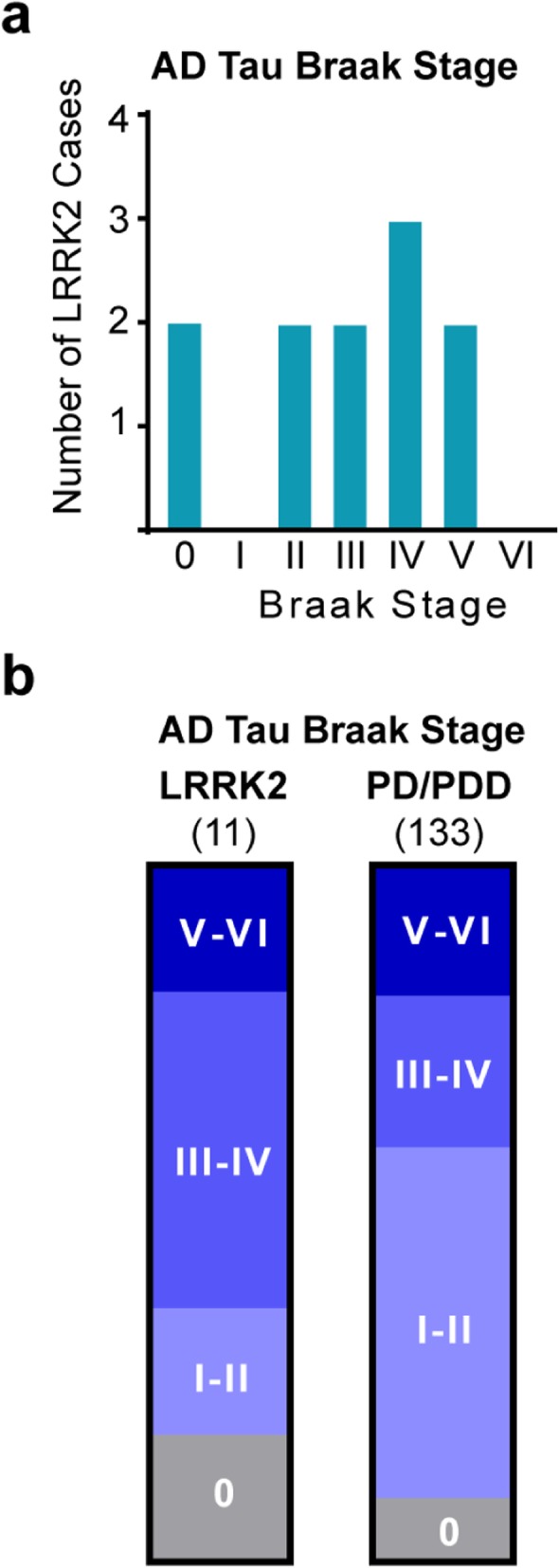
AD tau staging in *LRRK2* mutation carriers compared to idiopathic Lewy body disorders. **a** Tau pathology in *LRRK2* mutation carriers was categorized into classical Braak stages. One case, which did not have a clinical diagnosis of PD or PDD was excluded. **b** The proportion of *LRRK2* mutation carriers in each Braak stage of tauopathy is shown for 11 cases with *LRRK2* mutations (0: 18%; I-II: 18%; III-IV: 46%; V-VI: 18%) or 133 PD and PDD cases (0: 9%; I-II: 50%; III-IV: 22%; V-VI: 19%) published in a previous study [9]. The presence of tau pathology is similar between the two cohorts

AD neuropathology has recently been described as a major co-pathology in PD-PDD and is associated with progression to dementia [4, 9]. Therefore, we sought to determine whether the AD tau pathology observed in *LRRK2* PD-PDD is similar to that observed in iPD-PDD. Using the AD-selective tau antibody GT-38, *LRRK2* mutation carriers were staged using Braak AD tau stages 0-VI (Fig. 10a). Most *LRRK2* mutation carriers had mild to high levels of AD tau pathology, although none reached Braak stage VI. When compared to tau staging in 133 previously published PD-PDD cases, *LRRK2* PD had a similar prevalence of low to intermediate tau pathology (Braak I-IV) and high tau pathology (V-VI, Fig. 10b). The breakdown in stages I-II versus III-IV is slightly different in *LRRK2* mutation carriers versus iPD-PDD, although this could be partially due to the low number of *LRRK2* mutation carriers.

## Discussion

The presence of a familial disease that clinically mimics idiopathic disease can provide important mechanistic information about the pathogenesis of disease. The mutated protein may be involved in the biological pathway of disease and may be a therapeutic target for both familial and idiopathic forms of disease. In the case of PD, patients with or without *LRRK2* mutations seem to have similar disease onset and symptoms [18, 27]. However, whether or not *LRRK2* PD and iPD share the same pathological substrate has been unclear. It has been suggested that there may be two neuropathological classes of *LRRK2* PD [16, 28]. The most prominent of these is typical PD, with nigral degeneration and LBs; the second presents with nigral degeneration in the absence of LBs, but potentially with PSP-like tau pathology. The presence of LBs has been associated with progression to dementia in *LRRK2* mutation carriers, while cases without LBs manifest primarily motor phenotypes [14]. While AD tau has been described in *LRRK2* mutation carriers [13, 17, 18], the prevalence and extent of this pathology and its relationship with PSP-like tau has not been well-characterized. Therefore, in the current study, we sought to quantitatively characterize the level and extent of α-synuclein, tau, AD tau and Aβ pathologies in 12 *LRRK2* mutation carriers.

Similar to previous reports, nigral LB pathology was present in 64% (7/11) of our *LRRK2* mutation carriers. Remarkably, the 4 cases without nigral LBs also had no discernible Lewy pathology throughout all regions analyzed, clearly suggesting that phosphorylated α-synuclein is not a neuropathological substrate of disease in these patients. While the absence of α-synuclein pathology is rare in PD, we identified 9 “atypical PD” patients out of 109 total iPD patients in our database that had a similar nigral degeneration without LB pathology. Further, the tau pathology burden in the midbrain of LB-negative *LRRK2* mutation carriers was low, and was recognized by GT-38, suggesting that it is predominantly AD tau. Our data is therefore not consistent with tau acting as the neuropathological substrate of nigral neuron loss.

In contrast, AD-type tau is overall a prominent feature of *LRRK2* PD. This finding is corroborated by a recent report of 6 p.G2019S *LRRK2* mutation carriers which had variable LB pathology, but intermediate to high AD pathology in 83% (5/6) of the brains [29]. The level tau and Aβ pathology seen in this *LRRK2* PD cohort is more than would be expected for a non-demented cohort of individuals of a similar age [30, 31], but consistent with the prevalence of AD dementia in the population by this age [32]. Previous research has indicated that AD tau is a prominent feature of iPD and bridges the neuropathological space between PD, PDD, DLB, and AD, with higher AD pathology associated with a higher likelihood of progression to dementia [4, 9]. As in iPD-PDD, α-synuclein pathology burden is associated with both tau pathology and progression to dementia [9]. The AD tau pathology in *LRRK2* PD cases also follows similar Braak stages to those observed in iPD, suggesting that *LRRK2* PD is similar in pathological presentation to iPD.

Included in this study are two rare variants of unknown pathogenicity. The p.R793M variant was first identified in two families with a history of autosomal-dominant PD [33]. The pathogenicity of this variant was later called into question due to its detection in two healthy individuals in Norway, although the individuals were relatively young (59 and 61 years old) at the time [26]. The p.L1165P variant was described later in conjunction with the current p.R793M case as having abundant α-synuclein and tau pathology [24]. While little else is known about the p.L1165P variant pathologically, it has been shown to elevate kinase activity in an in vitro kinase assay [34], similar to p.G2019S and other variants [35]. Interestingly, these rare variants are unique among the cohort examined due to the high degree of tau pathology in the absence of any Aβ pathology. This makes these cases more likely to be primary tauopathies. However, the tau in these cases is also recognized by GT-38 and is present in areas indicative of AD pathology, so the tau in these cases may represent a continuum of AD that lacks Aβ pathology [36] or primary age-related tauopathy (PART) [37].

One limitation of the current study is the cohort size. While 11 PD-PDD patients with *LRRK2* mutations is a small group, it appears from our clinical and neuropathological assessment that these cases have captured the heterogeneity present in the larger *LRRK2* PD population, allowing us to understand general patterns of pathology. A second limitation of this study is the possible ascertainment bias from recruiting individuals from neurological disease centers. While this may lead to under sampling of neurologically normal cohorts, *LRRK2* genotyping was done in 94.9% of all individuals in the CNDR database where DNA was available. It will be interesting to see in the future if non-symptomatic *LRRK2* mutation carriers harbor any precursors of the neuropathology observed in our cases. One p.G2019S *LRRK2* mutation carrier without PD was included in our assessment, and had no apparent neuropathology. A final limitation is that the high prevalence of AD tau pathology could mask other, milder forms of tau pathology. Though we looked closely for non-AD tau in the substantia nigra, it is possible that non-AD tau is more prominent in other regions, including those that were not examined as part of the current study.

Beyond a neuropathological phenomenon, the prevalence of AD-type tau in *LRRK2* PD and iPD has important implications for modeling, diagnosis, and therapeutic treatment of PD. While much animal research has focused on investigating α-synuclein pathology in LRRK2 mutant animals, hyper-phosphorylated tau has been a consistent phenomenon observed in several LRRK2 mutant mouse lines [38–40]. Further, recent work has suggested that mutant LRRK2 may enhance spread of virally-expressed tau protein [41]. Our description of AD-type tau as a prominent pathology in *LRRK2* mutation carriers suggests that tau hyperphosphorylation may be an important component of *LRRK2*-related pathogenesis, and further investigation of the relationship between LRRK2 kinase activity and tau pathology in animal models is warranted.

Diagnosing PD and assessing therapeutic efficacy have been limited by the lack of reliable biomarkers for PD pathology. However, biomarkers are available for AD tau, including positron emission tomography (PET) imaging ligands [42] and cerebrospinal fluid tau and Aβ levels [43]. The presence of AD tau in iPD and *LRRK2* PD could serve as diagnostic and prognostic tools to segregate cases that will or will not progress to dementia, although further work is needed to validate tau as a biomarker in PD [44, 45]. Further, if tau pathology is modulated by the same biological mechanism as α-synuclein pathology, which may be the case for *LRRK2* PD, tau pathology could be used as a clinical trial endpoint measure of interest for therapy trials. Finally, if elevated LRRK2 kinase activity is directly modulating tau pathology, then LRRK2 inhibitors, which are already in clinical trials for PD, may modulate tau pathology in other primary tauopathies. However, whether LRRK2 kinase activity directly modulates tau pathology is not known, and future studies are warranted to investigate the role that LRRK2 plays in tau pathogenesis and whether LRRK2 inhibitors could serve as a potential therapeutic for tau pathology.

## Conclusion

Genetic forms of typically idiopathic diseases such as *LRRK2* PD provide a valuable opportunity to gain insight into the pathogenesis of disease. However, it is important to first understand the ways in which genetic PD is similar to or different from iPD. Our study demonstrates that the two disease entities are quite similar pathologically, including in the prevalence of comorbid AD-type pathologies. The remaining discrepancy between *LRRK2* PD and iPD is the lack of LBs or another pathological substrate of neuron loss in a subset of *LRRK2* mutation carriers. Future directions should focus on identifying the pathological substrate of neuron loss in these patients and understanding the relationship of *LRRK2* mutations to the development of tau pathology.

## Supplementary information

**Additional file 1: Figure S1.** Pathological α-synuclein and Aβ staining in tauopathy control brains. **Figure S2.** Prevalence of Aβ pathology in *LRRK2* mutation cases. **Figure S3.** Automated analysis enables quantitative pathology analysis. **Figure S4.** Regions with low GT-38 staining in *LRRK2* mutation carriers. **Figure S5.** Occurrence of co-pathologies. **Figure S6.** Tau and α-synuclein pathologies show minimal co-localization. **Figure S7.** Pathological correlates of clinical features by region in *LRRK2* mutation carriers.

**Additional file 2. Table S1.** Clinical and Pathological Data for Cases.

## Data Availability

The datasets used and/or analyzed during the current study are available from the corresponding author on reasonable request.

## Abbreviations

AD: Alzheimer’s disease
Aβ: Amyloid β
CA: Cornu Ammonis
CBD: Corticobasal degeneration
CNDR: Center for Neurodegenerative Disease Research
DLB: Dementia with Lewy bodies
FBS: Fetal bovine serum
iPD: Idiopathic PD
LBs: Lewy bodies
LRRK2: Leucine-rich repeat kinase
NFTs: Neurofibrillary tangles
PD: Parkinson’s disease
PDD: PD dementia
PET: positron emission tomography
PSP: Progressive supranuclear palsy
pSyn: pS129 α-synuclein

## References

[1] Postuma RB (2016). Abolishing the 1-year rule: how much evidence will be enough?. Mov Disord.

[2] Jellinger KA (2012). Neurobiology of cognitive impairment in Parkinson's disease. Expert Rev Neurother.

[3] McKeith IG (2017). Diagnosis and management of dementia with Lewy bodies: fourth consensus report of the DLB consortium. Neurology.

[4] Irwin DJ (2017). Neuropathological and genetic correlates of survival and dementia onset in synucleinopathies: a retrospective analysis. Lancet Neurol.

[5] Tsuboi Y, Uchikado H, Dickson DW (2007). Neuropathology of Parkinson's disease dementia and dementia with Lewy bodies with reference to striatal pathology. Parkinsonism Relat Disord.

[6] Hornykiewicz O (2002). Dopamine miracle: from brain homogenate to dopamine replacement. Mov Disord.

[7] Aarsland D (2003). Prevalence and characteristics of dementia in Parkinson disease: an 8-year prospective study. Arch Neurol.

[8] Braak H, Rub U, Del Tredici K (2006). Cognitive decline correlates with neuropathological stage in Parkinson's disease. J Neurol Sci.

[9] Irwin DJ (2012). Neuropathologic substrates of Parkinson disease dementia. Ann Neurol.

[10] Coughlin D (2019). Cognitive and pathological influences of tau pathology in Lewy body disorders. Ann Neurol.

[11] Healy DG (2008). Phenotype, genotype, and worldwide genetic penetrance of LRRK2-associated Parkinson's disease: a case-control study. Lancet Neurol.

[12] Lee AJ (2017). Penetrance estimate of LRRK2 p.G2019S mutation in individuals of non-Ashkenazi Jewish ancestry. Mov Disord.

[13] Poulopoulos M, Levy OA, Alcalay RN (2012). The neuropathology of genetic Parkinson's disease. Mov Disord.

[14] Kalia LV (2015). Clinical correlations with Lewy body pathology in LRRK2-related Parkinson disease. JAMA Neurol.

[15] Zimprich A (2004). Mutations in LRRK2 cause autosomal-dominant parkinsonism with pleomorphic pathology. Neuron.

[16] Spanaki C, Latsoudis H, Plaitakis A (2006). LRRK2 mutations on Crete: R1441H associated with PD evolving to PSP. Neurology.

[17] Ruffmann C (2012). Atypical tauopathy in a patient with LRRK2-G2019S mutation and tremor-dominant parkinsonism. Neuropathol Appl Neurobiol.

[18] Saunders-Pullman R (2018). Progression in the LRRK2-Asssociated Parkinson disease population. JAMA Neurol.

[19] Ross OA (2006). Lrrk2 R1441 substitution and progressive supranuclear palsy. Neuropathol Appl Neurobiol.

[20] Sanchez-Contreras M (2017). Study of LRRK2 variation in tauopathy: progressive supranuclear palsy and corticobasal degeneration. Mov Disord.

[21] Gibbons GS (2018). Detection of Alzheimer disease (AD)-specific tau pathology in AD and NonAD Tauopathies by immunohistochemistry with novel conformation-selective tau antibodies. J Neuropathol Exp Neurol.

[22] Gibbons GS (2019). Detection of Alzheimer's disease (AD) specific tau pathology with conformation-selective anti-tau monoclonal antibody in co-morbid frontotemporal lobar degeneration-tau (FTLD-tau). Acta Neuropathol Commun.

[23] Giasson BI (2006). Biochemical and pathological characterization of Lrrk2. Ann Neurol.

[24] Covy JP (2009). Clinical and pathological characteristics of patients with leucine-rich repeat kinase-2 mutations. Mov Disord.

[25] Team RC (2018). R: a language and environment for statistical computing.

[26] Toft M (2007). LRRK2 and Parkinson's disease in Norway. Acta Neurol Scand Suppl.

[27] Marras C (2011). Phenotype in parkinsonian and nonparkinsonian LRRK2 G2019S mutation carriers. Neurology.

[28] Wider C, Dickson DW, Wszolek ZK (2010). Leucine-rich repeat kinase 2 gene-associated disease: redefining genotype-phenotype correlation. Neurodegener Dis.

[29] Blauwendraat C (2019). Genetic analysis of neurodegenerative diseases in a pathology cohort. Neurobiol Aging.

[30] Bouras C (1994). Regional distribution of neurofibrillary tangles and senile plaques in the cerebral cortex of elderly patients: a quantitative evaluation of a one-year autopsy population from a geriatric hospital. Cereb Cortex.

[31] Nelson PT, Braak H, Markesbery WR (2009). Neuropathology and cognitive impairment in Alzheimer disease: a complex but coherent relationship. J Neuropathol Exp Neurol.

[32] Nelson PT (2012). Correlation of Alzheimer disease neuropathologic changes with cognitive status: a review of the literature. J Neuropathol Exp Neurol.

[33] Berg D (2005). Type and frequency of mutations in the LRRK2 gene in familial and sporadic Parkinson's disease*. Brain.

[34] Refai FS, Ng SH, Tan EK (2015). Evaluating LRRK2 genetic variants with unclear pathogenicity. Biomed Res Int.

[35] Steger M et al (2016) Phosphoproteomics reveals that Parkinson's disease kinase LRRK2 regulates a subset of Rab GTPases. Elife 5 10.7554/eLife.12813 PMC4769169 26824392

[36] Duyckaerts C (2015). PART is part of Alzheimer disease. Acta Neuropathol.

[37] Crary JF (2014). Primary age-related tauopathy (PART): a common pathology associated with human aging. Acta Neuropathol.

[38] Li Y (2009). Mutant LRRK2(R1441G) BAC transgenic mice recapitulate cardinal features of Parkinson's disease. Nat Neurosci.

[39] Yue M (2015). Progressive dopaminergic alterations and mitochondrial abnormalities in LRRK2 G2019S knock-in mice. Neurobiol Dis.

[40] Schapansky J (2018). Familial knockin mutation of LRRK2 causes lysosomal dysfunction and accumulation of endogenous insoluble alpha-synuclein in neurons. Neurobiol Dis.

[41] Nguyen APT (2018). G2019S LRRK2 enhances the neuronal transmission of tau in the mouse brain. Hum Mol Genet.

[42] Johnson KA (2016). Tau positron emission tomographic imaging in aging and early Alzheimer disease. Ann Neurol.

[43] Niemantsverdriet E (2017). Alzheimer's disease CSF biomarkers: clinical indications and rational use. Acta Neurol Belg.

[44] Jimenez-Jimenez FJ (2014). Cerebrospinal fluid biochemical studies in patients with Parkinson's disease: toward a potential search for biomarkers for this disease. Front Cell Neurosci.

[45] Magdalinou N, Lees AJ, Zetterberg H (2014). Cerebrospinal fluid biomarkers in parkinsonian conditions: an update and future directions. J Neurol Neurosurg Psychiatry.

