# Correction to: Alzheimer’s disease tau is a prominent pathology in LRRK2 Parkinson’s disease

**DOI:** 10.1186/s40478-024-01819-7

**Published:** 2024-06-28

**Authors:** Michael X. Henderson, Medha Sengupta, John Q. Trojanowski, Virginia M. Y. Lee

**Affiliations:** Department of Pathology and Laboratory Medicine, Institute on Aging and Center for Neurodegenerative Disease Research, University of Pennsylvania School of Medicine, 3600 Spruce St, 3rd Floor Maloney, Philadelphia, PA 19104 USA

**Correction to: Acta Neuropathologica Communications (2019) 7:183** 10.1186/s40478-019-0836-x

Following publication of the original article [1], the authors identified an error in Figure 2. Figure 2 had a duplicated image panel.

The duplicated images represented a figure design error and do not impact interpretation of the manuscript.

The incorrect Figure 2:

**Fig. 2:**
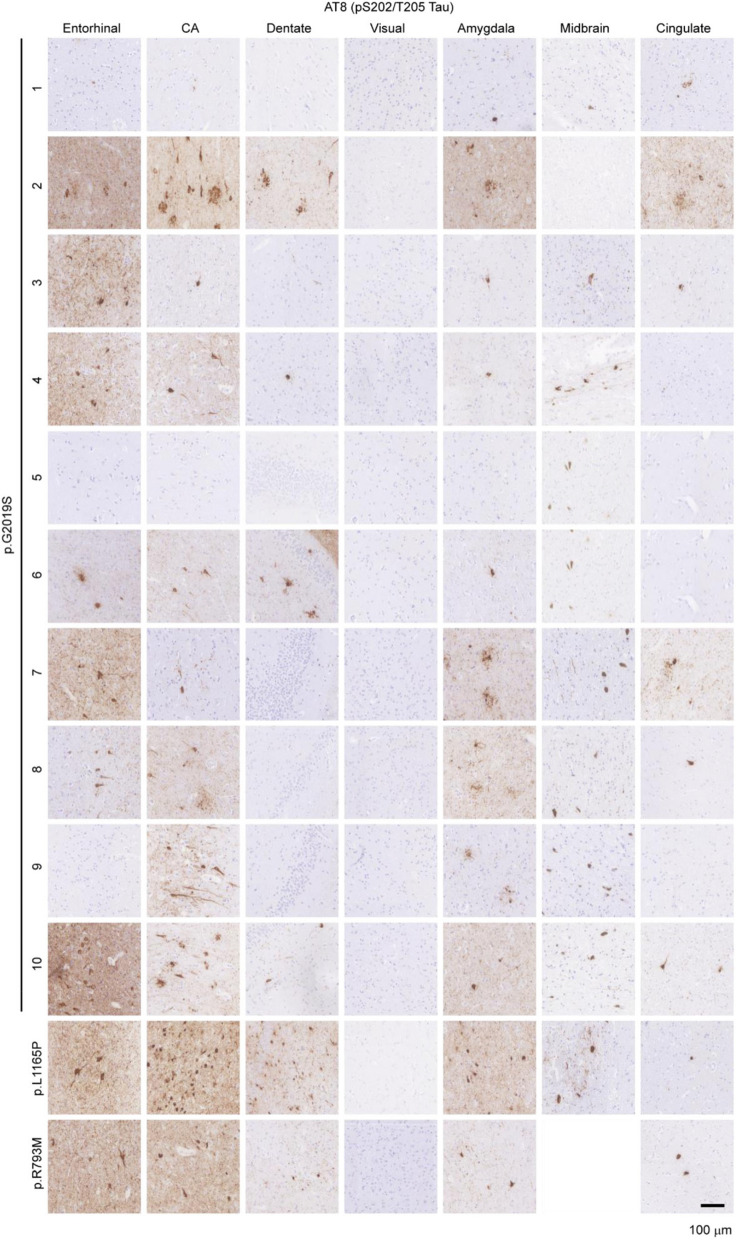
Prevalence of tau pathology in *LRRK2* mutation carriers. Seven brain regions from 12 individuals carrying *LRRK2* mutations were evaluated by staining for pathological pS202/T205 tau (AT8). Of the 12 cases, 10 showed prominent pathological tau, including in the hippocampus, while 2 cases had minimal pathological tau present. Scale bar = 100 µm

The correct Figure 2:

**Fig. 2:**
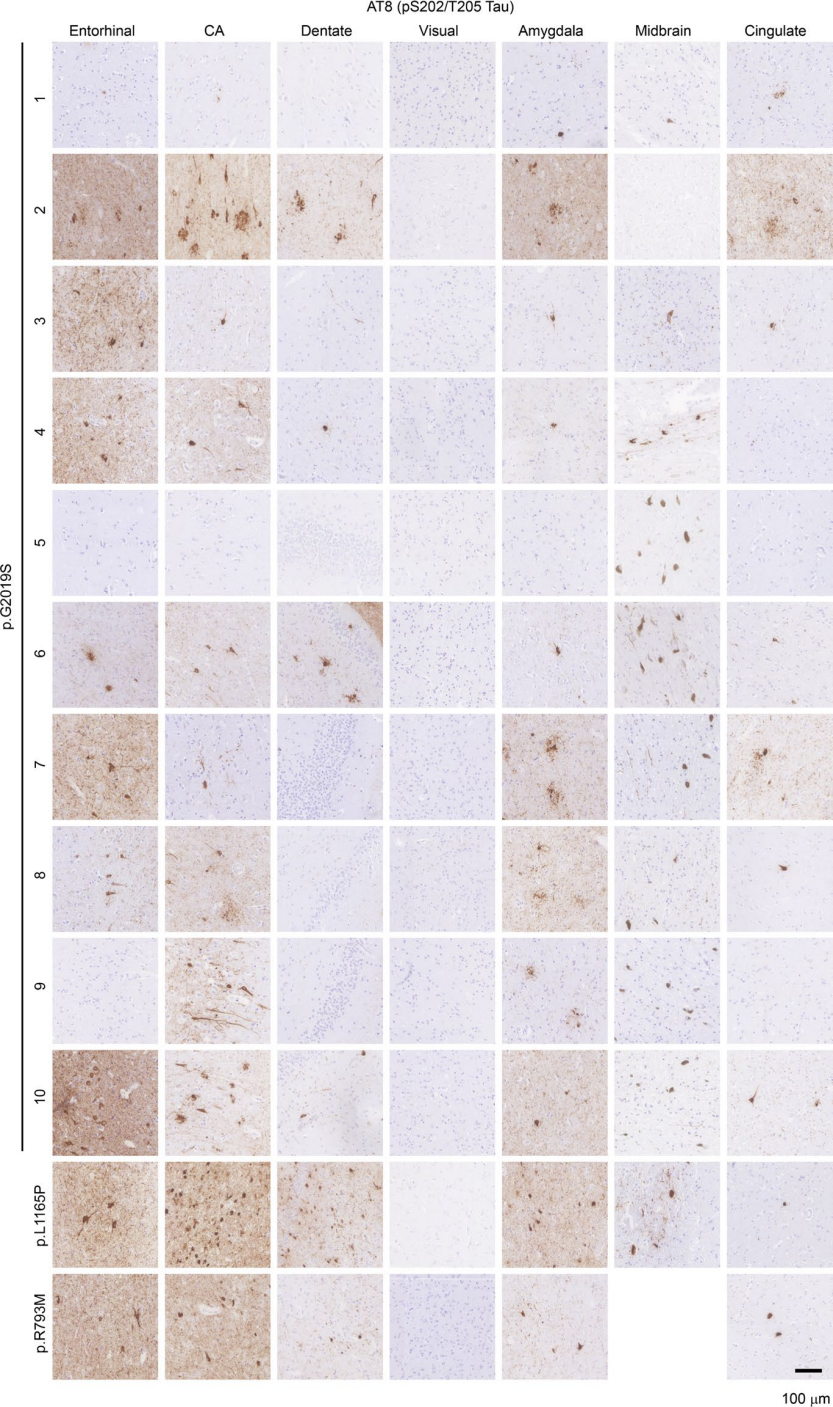
Prevalence of tau pathology in *LRRK2* mutation carriers. Seven brain regions from 12 individuals carrying *LRRK2* mutations were evaluated by staining for pathological pS202/T205 tau (AT8). Of the 12 cases, 10 showed prominent pathological tau, including in the hippocampus, while 2 cases had minimal pathological tau present. Scale bar = 100 µm

The correct Figure 2 has been indicated in this correction article and the original article [1] has been corrected.
