# Supplementary material for: Alzheimer’s disease tau is a prominent pathology in LRRK2 Parkinson’s disease
Source: Acta Neuropathol Commun. 2019 Nov 16;7:183. doi: 10.1186/s40478-019-0836-x (PMC6858668; doi:10.1186/s40478-019-0836-x)
Supplement: Supplementary file 1 — Additional file 1: Figure S1. Pathological α-synuclein and Aβ staining in tauopathy control brains. Figure S2. Prevalence of Aβ pathology in LRRK2 mutation cases. Figure S3. Automated analysis enables quantitative pathology analysis. Figure S4. Regions with low GT-38 staining in LRRK2 mutation carriers. Figure S5. Occurrence of co-pathologies. Figure S6. Tau and α-synuclein pathologies show minimal co-localization. Figure S7. Pathological correlates of clinical features by region in LRRK2 mutation carriers. [file 40478_2019_836_MOESM1_ESM.docx]

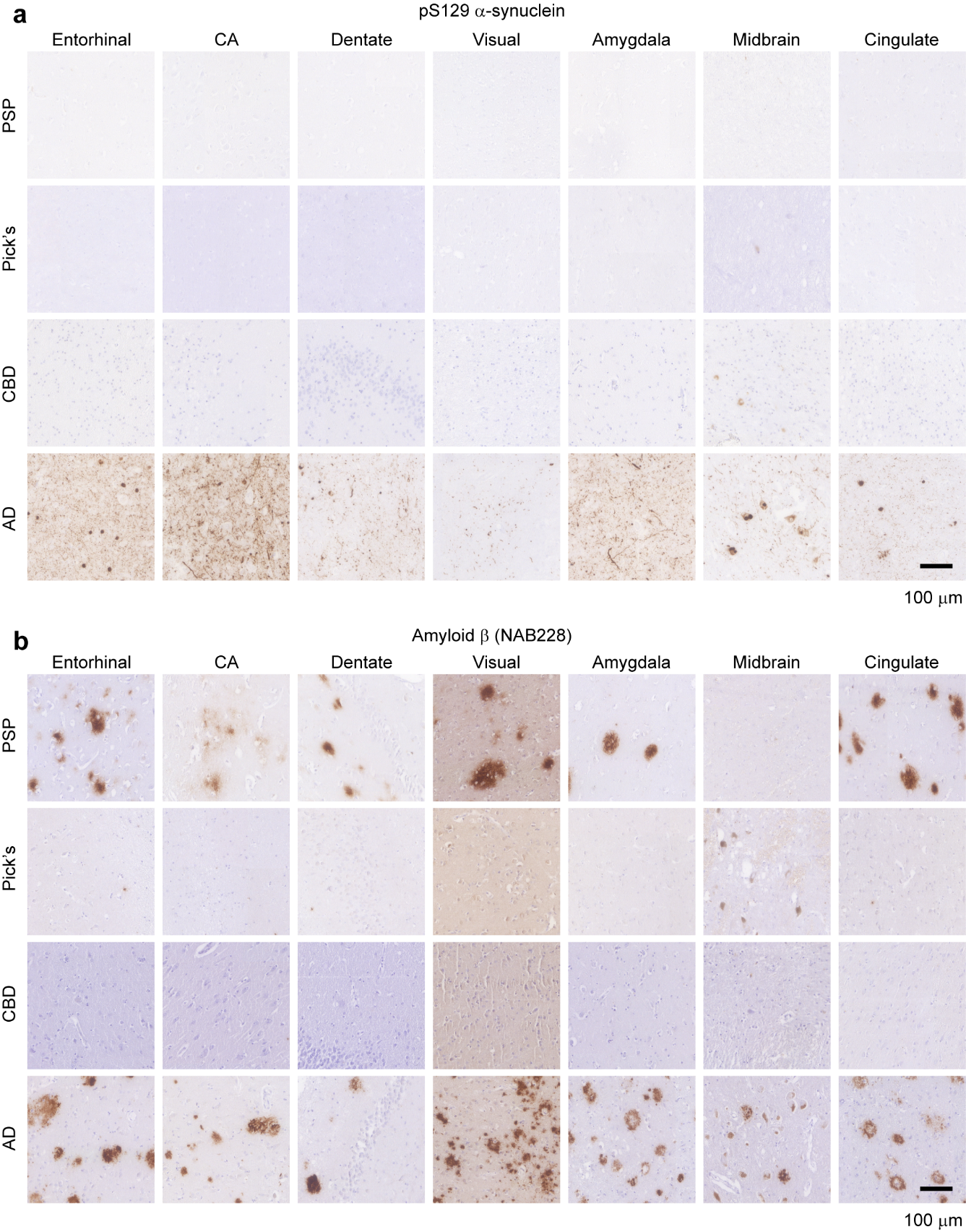
**Supplementary Figures**

**Fig. S1 Pathological α-synuclein and Aβ staining in tauopathy control brains. a** A pS129 α-synuclein antibody (EP1536Y) was used to stain pathological α-synuclein in 7 regions from 4 different tauopathy brains, progressive supranuclear palsy (PSP), Pick’s disease, corticobasal degeneration (CBD) and Alzheimer’s disease (AD). **b** Aβ was stained in adjacent sections to those stained in panel (**a**). Scale bar = 100 μm.


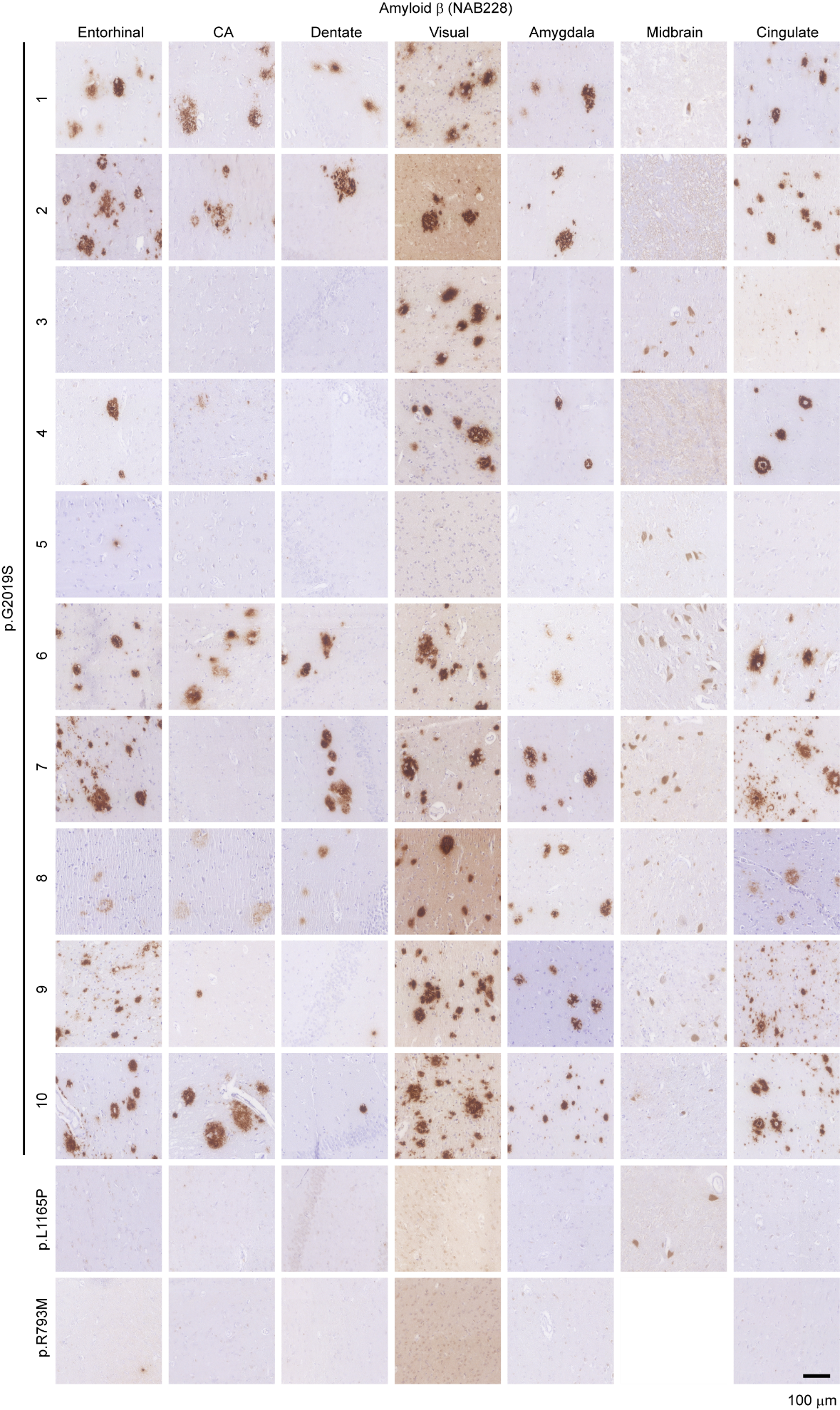


**Fig. S2 Prevalence of Aβ pathology in *LRRK2* mutation cases.** Seven brain regions from 12 individuals carrying *LRRK2* mutations were evaluated by staining for pathological Aβ (NAB228). Of the 12 cases, 9 showed prominent pathological Aβ in at least the visual cortex. Three cases, including the two rare variants showed no apparent Aβ pathology. Scale bar = 100 μm.

**
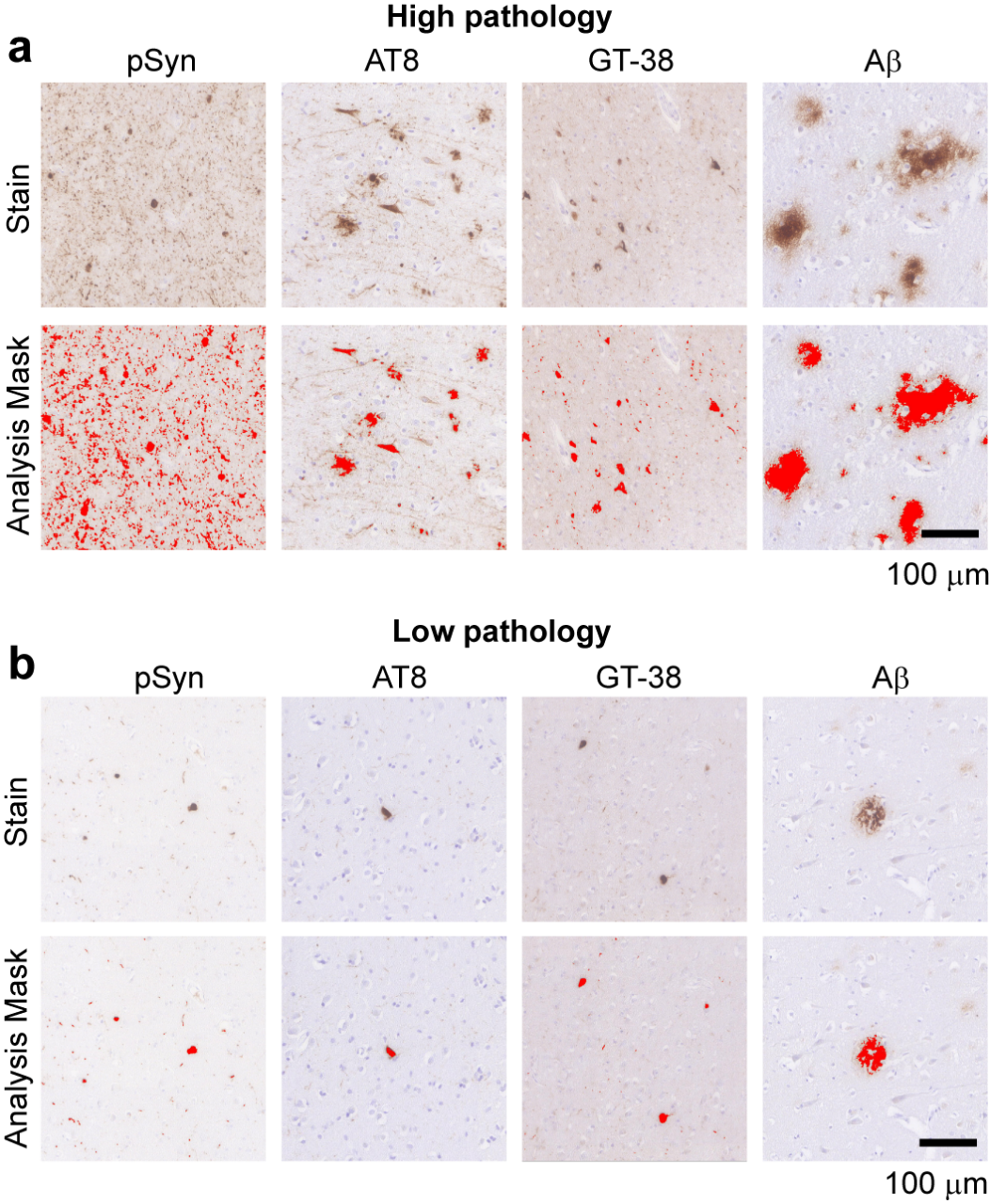
**

**Fig. S3 Automated analysis enables quantitative pathology analysis.** Automated analysis of pathology was based on empirically-optimized optical density thresholds of stained tissue. **a** Regions with high pSyn, AT8, GT-38 or Aβ pathology are shown with or without an analysis overlay (red) which was used to quantify the percentage of area occupied by pathology. **b** Regions with low pSyn, AT8, GT-38 or Aβ pathology are shown for comparison. Thresholds were optimized so that even low amounts of pathology could be quantified without the inclusion of background staining. Scale bar = 100 μm.

**
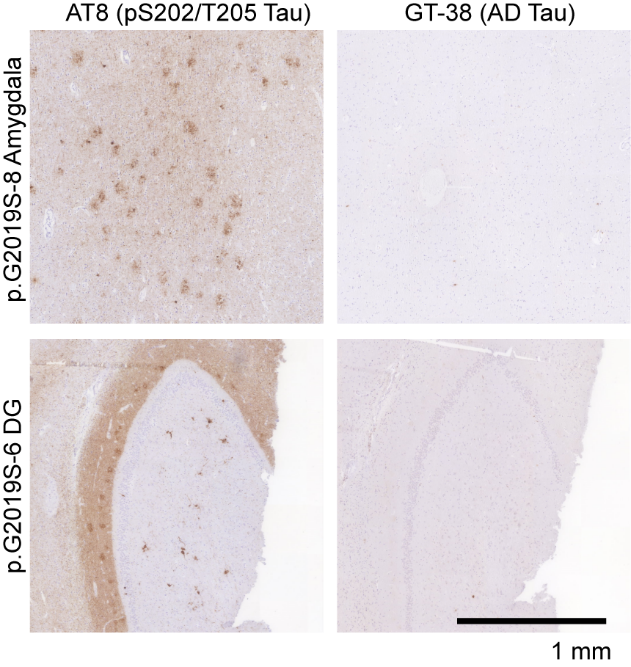
**

**Fig. S4 Regions with low GT-38 staining in *LRRK2* mutation carriers.** While most regions in *LRRK2* mutation carriers had a high correspondence between the AT8 and GT-38 percentage area stained, there were several regions that had substantially higher AT8 than GT-38 stain. Two such regions are displayed here. Each region had very abundant neuritic tau pathology stained by AT8, but relatively little stain by GT-38. Scale bar = 1 mm.

**
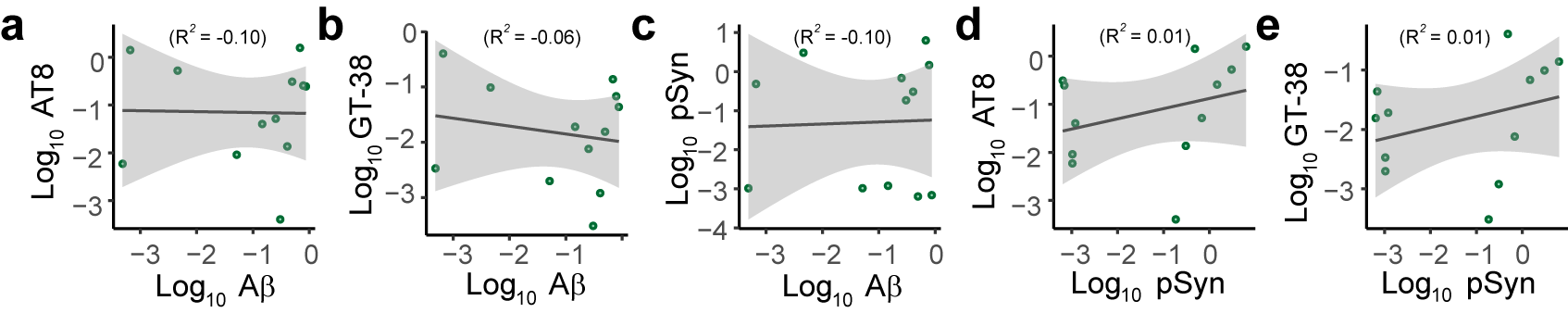
**

**Fig. S5 Occurrence of co-pathologies.** Many regions of the brain had multiple pathologies co-occurring. To understand whether there was an association of the different pathologies to each other, each different pathology was plotted against the other by individual cases (**a-e**). While Aβ burden showed no relationship to tau or α-synuclein burden (**a** Aβ x AT8: *R^2^_adj_* = -0.10, p = 0.9491; **b** Aβ x GT-38: *R^2^_adj_* = -0.06, p = 0.5728; **c** Aβ x pSyn: *R^2^_adj_* = -0.10, p = 0.9056;), pSyn pathology was mildly associated with tau pathology (**i** pSyn x AT8: *R^2^_adj_* = 0.01, p = 0.3199; **i** pSyn x GT-38: *R^2^_adj_* = 0.01, p = 0.3174), suggesting that the two pathology may influence each other or may both be influenced by a common factor.

**
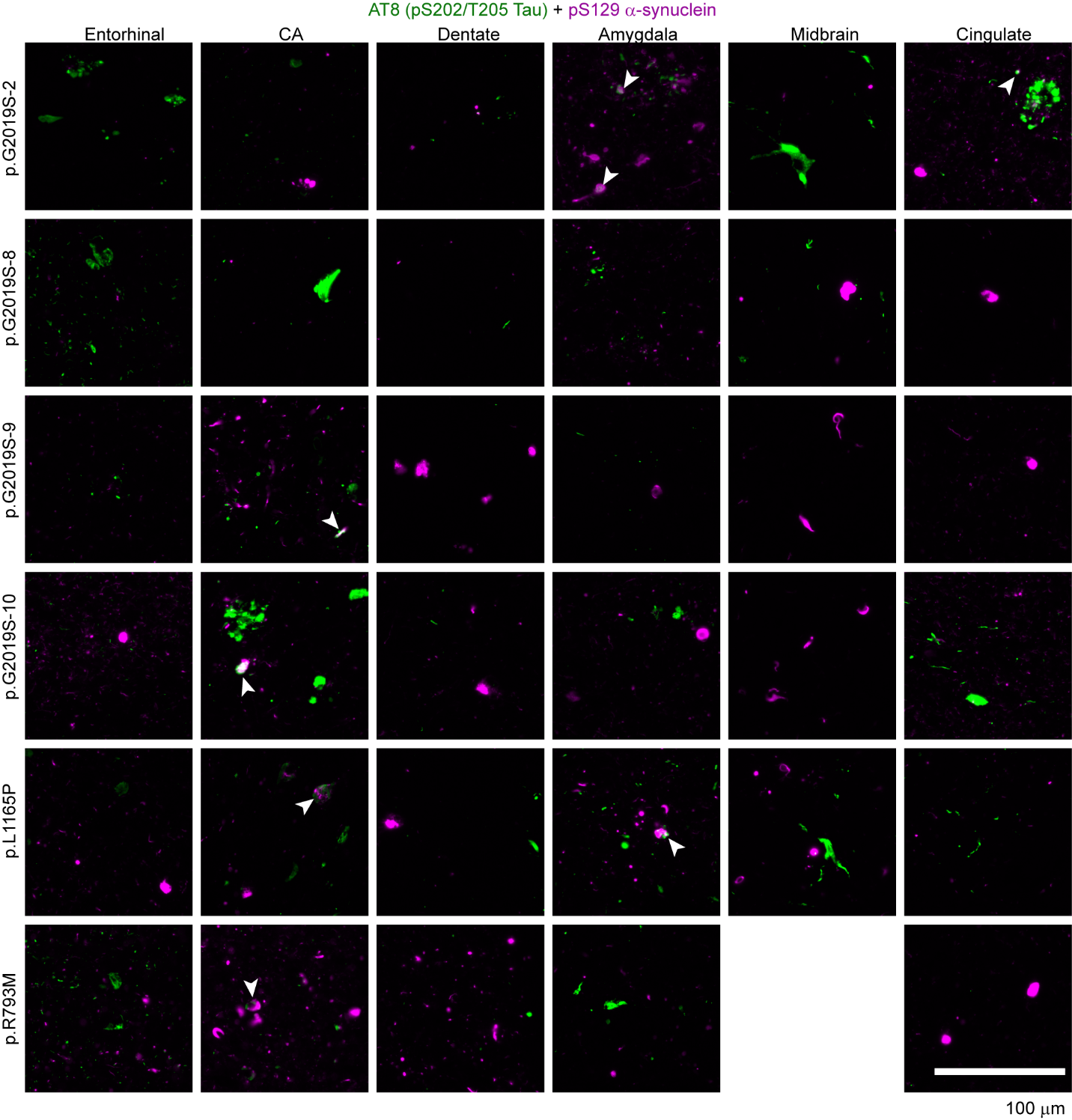
Fig. S6 Tau and α-synuclein pathologies show minimal co-localization.** To examine whether tau and α-synuclein pathology occur in the same neurons, sections from *LRRK2* mutation carriers that were positive for both tau and α-synuclein pathology were labeled with AT8 (green) for tau pathology and pS129 α-synuclein (magenta) for LB and LN pathology. Almost all regions examined contained both pathologies, but co-occurrence of both pathologies in the same cell was rare and is noted by white arrowheads. Scale bar = 100 μm.

**
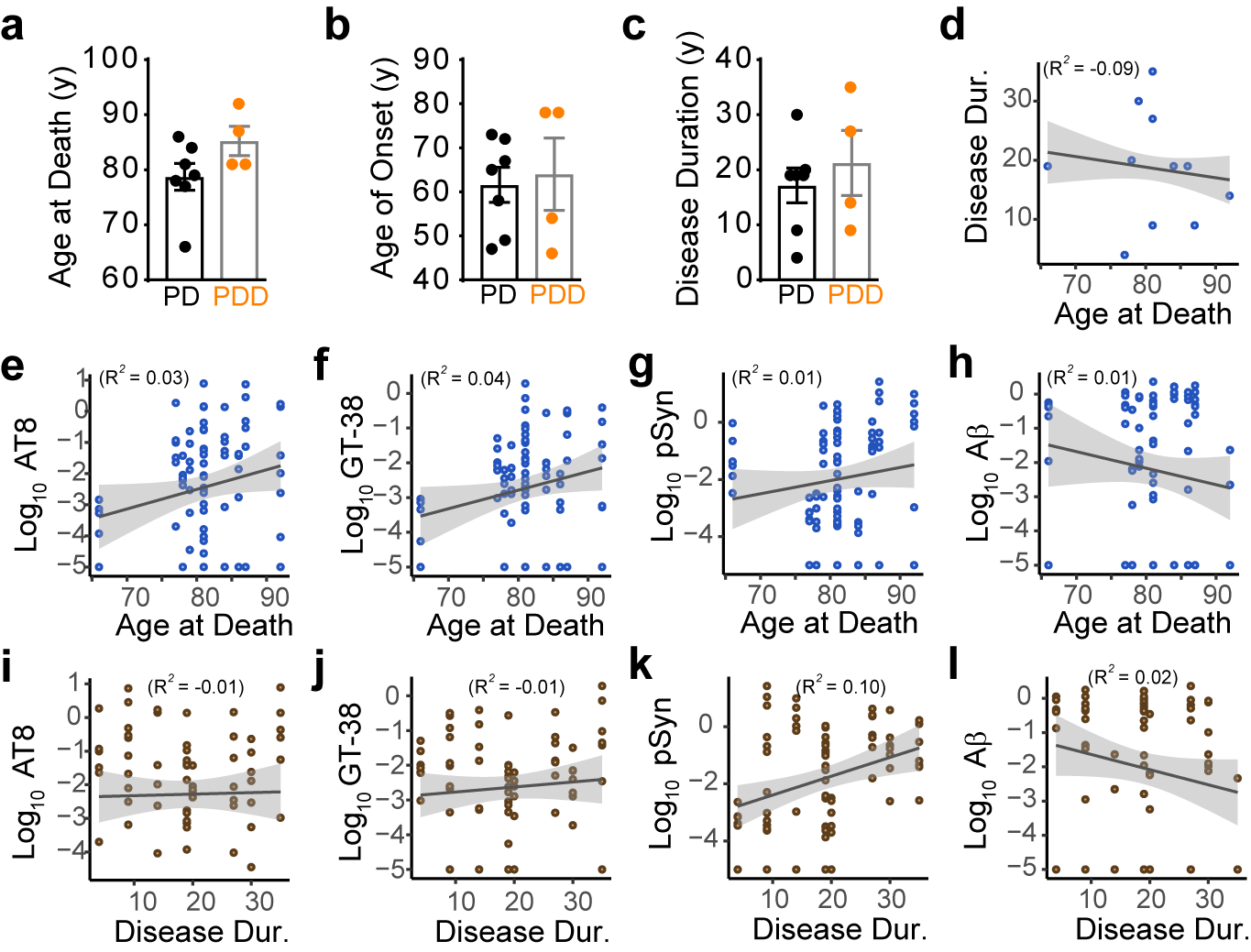
**

**Fig. S7 Pathological correlates of clinical features by region in *LRRK2* mutation carriers. a-c** Comparisons of clinical features in *LRRK2* mutation carriers that had either PD or PD with dementia (PDD). Of the 12 *LRRK2* mutation carriers, 11 of them were characterized clinically as having PD, or having PD followed by dementia. One case had a clinical history of schizophrenia, not PD, and so was removed from this analysis. Age at death (**a**), age of onset (**b**) and disease duration (**c**) was not significantly different in individuals with dementia. **a-c** Unpaired t-tests: Age at Death: p = 0.1222; Age of Onset: p = 0.7694; Disease Duration: p = 0.5165. **d** Age at death and disease duration showed no correlation (*R^2^_adj_* = -0.09, p = 0.7109). **e-h** Log_10_ pathology levels for each region were plotted against age at death. AT8 and GT-38 showed the best correlation with age. Lines represent linear regression line of best-fit and shaded area is the 95% confidence interval (AT8: *R^2^_adj_* = 0.03, p = 0.05059; GT-38: *R^2^_adj_* = 0.04, p = 0.04254; pSyn: *R^2^_adj_* = 0.01, p = 0.1595, Aβ: *R^2^_adj_* = 0.01, p = 0.2018). **i-l** Log_10_ pathology levels for each region were plotted against disease duration. pSyn levels showed the best correlation with disease duration (AT8: *R^2^_adj_* = -0.01, p = 0.8425; GT-38: *R^2^_adj_* = -0.01, p = 0.4399; pSyn: *R^2^_adj_* = 0.10, p = 0.0030, Aβ: *R^2^_adj_* = 0.02, p = 0.09108).
